# Nitrogen-induced metabolic changes and molecular determinants of carbon allocation in *Dunaliella tertiolecta*

**DOI:** 10.1038/srep37235

**Published:** 2016-11-16

**Authors:** Kenneth Wei Min Tan, Huixin Lin, Hui Shen, Yuan Kun Lee

**Affiliations:** 1 Department of Microbiology and Immunology, Yong Loo Lin School of Medicine, National University of Singapore, 117545, Singapore

## Abstract

Certain species of microalgae are natural accumulators of lipids, while others are more inclined to store starch. However, what governs the preference to store lipids or starch is not well understood. In this study, the microalga *Dunaliella tertiolecta* was used as a model to study the global gene expression profile regulating starch accumulation in microalgae. *D. tertiolecta*, when depleted of nitrogen, produced only 1% of dry cell weight (DCW) in neutral lipids, while starch was rapidly accumulated up to 46% DCW. The increased in starch content was accompanied by a coordinated overexpression of genes shunting carbon towards starch synthesis, a response not seen in the oleaginous microalgae *Nannochloropsis oceanica*, *Chlamydomonas reinhardtii* or *Chlorella vulgaris*. Genes in the central carbon metabolism pathways, particularly those of the tricarboxylic acid cycle, were also simultaneously upregulated, indicating a robust interchange of carbon skeletons for anabolic and catabolic processes. In contrast, fatty acid and triacylglycerol synthesis genes were downregulated or unchanged, suggesting that lipids are not a preferred form of storage in these cells. This study reveals the transcriptomic influence behind storage reserve allocation in *D. tertiolecta* and provides valuable insights into the possible manipulation of genes for engineering microorganisms to synthesize products of interest.

In recent years, microalgae have emerged as a promising source for the production of renewable biofuels, as well as additional feedstock for food, feed, and chemicals^1^,^2^,^3^. Compared to terrestrial plants, microalgae boast higher photosynthetic efficiency and are able to channel most of their energy into cell division, allowing for rapid biomass production rates^2^,^3^,^4^. In addition, they can be cultivated in wastewater and saltwater systems which will not directly compete with resources necessary for agricultural food production^5^,^6^. When cultivated under stressful conditions such as nitrogen depletion (N-depletion), photosynthetic microalgae alter their metabolism to redirect energy towards the production and accumulation of energy-rich storage compounds such as starch and lipids^7^,^8^,^9^, but at the cost of diminished growth. This ability enables the microalgae to survive adverse environmental changes as the energy deposits can be easily mobilized when growth conditions are restored^10^. Some microalgae species are oleaginous and are capable of producing up to 60% of neutral lipids (i.e. triacylglyerols; TAGs) per gram of dry weight^11^, while non-oleaginous microalgae specialize in accumulating carbohydrates^12^. What influences one microalgae to store lipids and another to preferentially store starch is poorly understood.

Traditionally, *Nannochloropsis oceanica* strains are favored for studying lipid accumulation^13^,^14^,^15^, and the freshwater microalgal species *Chlamydomonas reinhardtii*^16^,^17^ and *Chlorella vulgaris*^8^,^18^,^19^ are most commonly studied for starch accumulation. The latter two species have thus far been cultivated mixotrophically or heterotrophically with the addition of organic carbon such as acetate and glucose^20^. However, there are significant drawbacks in this strategy as it would expose the culture to higher risks of contamination and is not ideal for microalgal CO_2_ fixation^21^. *Dunaliella tertiolecta* – a non-model flagellate microalga belonging to the phylum Chlorophyta – is one of the most promising candidates for industrial use as it is among the top microalgal biomass-producers^12^,^22^, is halotolerant and can be grown in high salinity environments (up to 4 M NaCl) such as saltwater, wastewater or brackish water^23^, which could exclude contaminating species and would not compete with freshwater sources. Importantly, it is a robust species that is able to maintain high growth rates in a wide range of pH, temperature and light, and contain relatively high lipid content^2^,^24^,^25^. Unlike most microalgae *D. tertiolecta* do not have a cell wall, making cell lysis much less energy intensive while auto-flocculation drastically reduces energy required for harvesting^26^. A shortcoming is that its genome is unsequenced, which largely limits our understanding of its behavior at the molecular level. In 2011, Rismani-Yazdi *et al.* provided a preliminary annotation of available genes and putative pathways in *D. tertiolecta* based on sequence similarity between *Chlamydomonas reinhardtii* and *Volvox carteri*^27^, but did not compare the transcriptomic response between physiological states. Recently, several new transcriptomic studies of *Dunaliella* species have been conducted^28^,^29^,^30^. Yao *et al.* created a draft database for annotation of RNA-seq data from *D. tertiolecta* and described pathway analyses of a random mutant with enhanced lipid production^28^. Similarly, Shin *et al.* independently annotated the transcriptome of *D. tertiolecta* and studied possible genes related to growth limitation during N-depletion^29^.

In this study, we aim to determine the underlying changes in gene expression which may influence storage product accumulation in *D. tertiolecta*, and thus establish a transcriptomic basis for the preference of *D. tertiolecta* to store one product over another. We tracked for dynamic changes of storage components including starch, glycerol, neutral lipids and total fatty acids, and compared the transcriptome profile of the cell in the exponential and stationary phases after nitrogen has been depleted in the media. Our investigations into the transcriptome profile via RNA-seq uncovered key characteristics linked to increased activity in the central carbon metabolism (CCM) pathways such as the tricarboxylic acid (TCA) cycle, glycolysis and the oxidative pentose phosphate pathway (OPPP) that may contribute to carbon flux and starch synthesis. Finally, we compared our findings with microalgae species of varying oleaginicity to better understand the underlying differences that determine the preferred production of starch and/or lipids.

## Results

### Physiological response of *D. tertiolecta* under Nitrogen depletion

**Figure 1:**
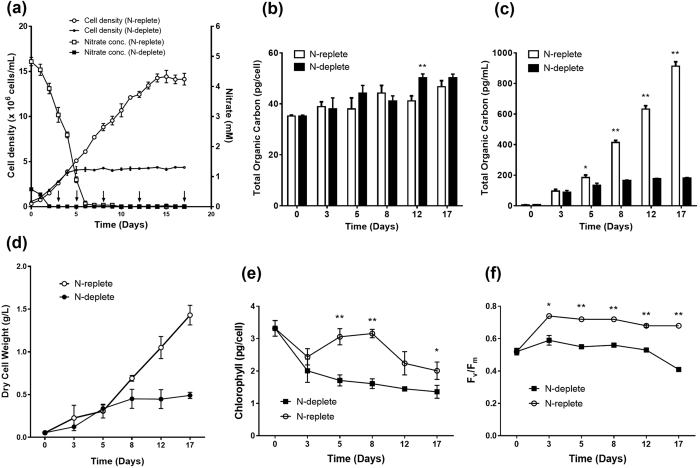
Physiological parameters of *D. tertiolecta* under nitrogen depletion. *D. tertiolecta* was cultured in 50-mL batch cultures supplemented with 5% CO_2_. Cell growth and media nitrogen concentration (**a**) was measured every day. Total organic carbon (**b,c**) and dry cell weight (**d**), chlorophyll (**e**) and photosynthetic yield (**f**) were measured at different time points (Day 0, 3, 5, 8, 12, 17) to illustrate the different phases of growth. Error bars represent standard deviations from three independent biological replicates. Asterisks indicate statistically significant differences between N-replete and N-deplete samples after two-tailed t-tests (*p value ≤ 0.05; **p value ≤ 0.01).

In the *Dunaliella* genus which dominates list of top strains for carbohydrate production, *D. tertiolecta* is one of the most researched species due to its ease of culture, tolerance to varied environmental conditions, fast growth rate, high accumulation of storage compounds per dry weight (protein, starch, lipids), and overall biomass productivity^12^,^31^, making it an attractive microalgal feedstock for commercial mass cultivation^32^. *D. tertiolecta* strain UTEX LB 999 was grown in ATCC-1174 DA medium with an initial nitrate concentration of 5 mM and 0.5 mM for N-replete and N-deplete cultures respectively. From day 1 to 15, cell density for N-replete cultures increased from 0.75 × 10^6^ to 14.4 × 10^6^ cells/mL, before dropping to 13.88 × 10^6^ cells/mL (Fig. 1a), suggesting that cell division had ceased. On the other hand, N-deplete cultures could only reach a maximum cell density of 4.28 × 10^6^ cells/mL, on day 8. The nitrate concentrations in the culture medium was depleted after 3 days for N-deplete cells, and 9 days for N-replete cells (Fig. 1a), but cell growth continued for N-replete cells, likely driven by intracellular nitrogen stores. For the purpose of this study, cells were harvested for metabolite analyses at days 3, 5, 8, 12, and 17 to capture the passage through exponential and stationary growth phases.

Although cell division has slowed for N-deplete cells from the fourth day onwards, the increase in dry weight (Fig. 1d) was largely due to the accumulation of storage compounds such as starch and glycerol, as the cells continue to accumulate organic carbon (Fig. 1b,c). Over the course of the experiment, N-depletion also led to reduced total chlorophyll content (Fig. 1e) and photosynthetic yield (F_v_/F_m_) (Fig. 1f). Although there was a decrease in chlorophyll content for both N-replete and N-deplete cultures on day 3, N-replete cultures subsequently recovered chlorophyll levels to the original state (i.e. Day 0) during the exponential growth phase, while those of N-deplete cultures continue to fall (Fig. 1e). The health of photosystem II as determined by F_v_/F_m_, held steady at around 0.68–0.74 for N-replete cultures. On the other hand, N-deplete cultures experience a drop in photosynthetic yield from 0.59 to 0.41, indicative of cell stress and possible damage to photosystem II (Fig. 1f).

### Accumulation pattern of storage compounds upon N-depletion

**Figure 2:**
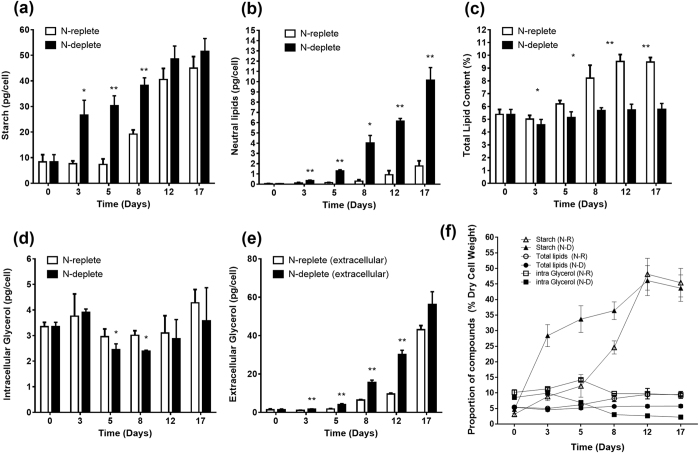
Effects of nitrogen depletion on storage product accumulation in *D. tertiolecta*. The starch (**a**), neutral lipids (**b**), total lipids (**c**), intracellular glycerol (**d**) and extracellular glycerol (**e**) content in *D. tertiolecta* cells under nitrogen-replete and nitrogen-deplete conditions in different phases of cell growth. Proportion of starch, total lipids and intracellular glycerol are presented as a percentage of dry cell weight (**f**). Error bars represent standard deviations from three independent biological replicates. Asterisks indicate statistically significant differences between N-replete and N-deplete samples after two-tailed t-tests (*p value ≤ 0.05; **p value ≤ 0.01).

**Figure 3:**
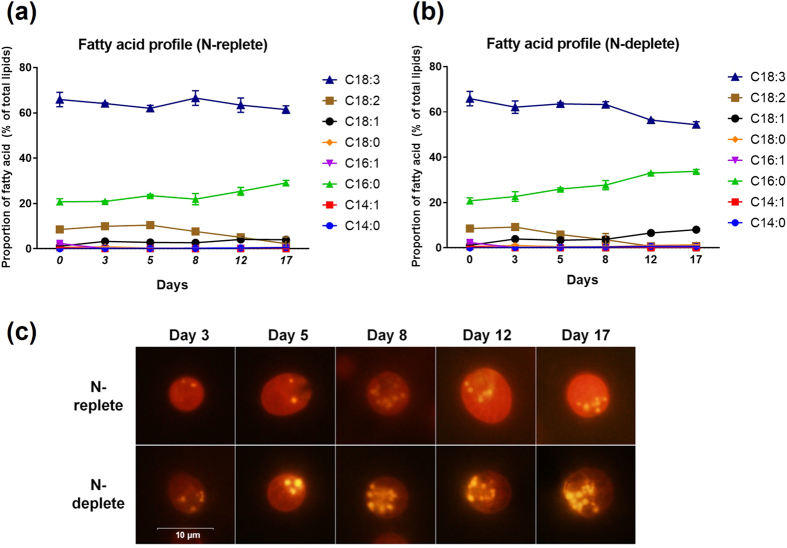
Fatty acid profiles and detection of neutral lipids by Nile red staining of *D. tertiolecta* lipids. Proportion of the major types of fatty acids (**a,b**) are expressed as percentages of total lipids. After staining with the nonpolar lipid fluorophore Nile red, cells were examined by fluorescence microscopy which showed the formation of lipid droplets corresponding with the state of nitrogen depletion (**c**). Yellow fluorescence reflects the staining of neutral lipids, which form lipid droplets. Red fluorescence corresponds to background chlorophyll auto-fluorescence.

Lipid, starch and glycerol synthesis pathways share common carbon precursors fed from the glycolytic pathway, but the regulation of carbon allocation into these routes is not well understood^33^,^34^. Under the favorable condition where nitrogen is available, N-replete *D. tertiolecta* maintained basal levels of starch (7–8 pg/cell) during the first 5 days of cultivation; consequently, when nitrogen is depleted, it increasing to 19 pg/cell on day 8 (Fig. 2a). In contrast, N-deplete cultures had starch rapidly accumulated on the third day (27 pg/cell), and the starch content of the latter remained higher during the entire process. Interestingly, starch was accumulated much earlier and in greater absolute amounts compared to lipids (neutral lipids and total lipids) (Fig. 2b,c). Notably, despite a large increase in neutral lipids (Figs 2b and 3c), N-depletion did not result in a corresponding increase in total lipids (Fig. 2c). On the contrary, it led to a cessation in its production (5.8% in N-deplete vs. 9.5% in N-replete on Day 17). Intracellular glycerol was relatively constant for both cultures throughout the experiment (Fig. 2d), with only extracellular glycerol showing marked increase, especially by N-deplete cells (Fig. 2e). This is consistent with findings in current literature implicating the continued release of glycerol by *D. tertiolecta* into the culture medium^35^,^36^. The amount of extracellular glycerol produced was able to reach higher quantities as it is not restricted by cell volume, enabling it to deliver microgram levels per cell not achievable with intracellular glycerol, starch or lipids.

When presented on a percentage dry cell weight (DCW) basis, similar trends were observed with subtle differences. Starch content of N-deplete cells peaked sharply on day 3 (28% DCW) compared to N-replete cells where it moderately increased until the media is deprived of nitrogen on day 8 (25% DCW) (Fig. 2f). Intracellular glycerol accounted for 9–14% of DCW in N-replete cells and N-deplete cells. However, in the latter stages (Day 8–17), intracellular glycerol representation in N-deplete cells dropped to 2–3% DCW as the DCW increased while intracellular glycerol content remained the same. Total lipid production was greatly attenuated in N-deplete cultures, accounting for 5.8% DCW on day 17, which is 39% less than the N-replete culture (Fig. 2f). There were minimal changes to the fatty acid (FA) composition between either cultures; C18:3 is the most abundant FA in *D. tertiolecta*, making up to 66% of its profile (Fig. 3a,b). The proportion of C16:0, however, appears to increase over time particularly for cells experiencing N-depletion, while those of C18:2 and C18:3 levels declined.

### Analysis of gene expression by transcriptomics

The growth of *D. tertiolecta* in either cultures presented two distinctive phases: Exponential and Stationary (Fig. 1a). To assess the transcriptional regulation of selective carbon partitioning, we carried out transcriptome analyses at 2 times points representing N-deplete exponential phase (N-replete vs. N-deplete; both in Day 3 exponential phase) and stationary phase (N-replete exponential vs. N-deplete Day 5 stationary phase). Gene expression was expressed as fold change relative to N-replete samples. Out of all the annotated genes for Day 3 (D3) and Day 5 (D5) samples, we filtered for significantly expressed genes using a False discovery rate (FDR)-corrected p-value ≤ 0.05. This translated to 3,962 and 1,234 significantly expressed genes for D3 and D5, accounting respectively for ~17 and 6% of all genes annotated (Table S2). Among this filtered set of genes, 96% (D3) and 89% (D5) of genes were over/under-expressed (≤−2x or ≥ 2x fold-change) in N-deplete samples relative to N-replete samples.

### Functional annotation and enrichment of differentially expressed genes

Gene Ontology (GO) enrichment of the differentially expressed genes showed that in D3, upregulated genes form the majority of categories while in D5 most genes were downregulated (Figs S2, S3). Nitrogen compound metabolic process was upregulated in both D3 and D5, an indication that the cells were increasing capacity for utilizing nitrogen in response to the nutrient’s depletion. The top 10 most highly expressed or repressed genes were related to nitrogen-scavenging or photosynthesis (Table S3). For instance, THB1, a truncated hemoglobin highly expressed in the presence of nitric oxide^37^, was repressed by almost 300-fold. Likewise, three subunits of the urea active transporter were upregulated between 52- and 72-fold, representing one of the many enzymes whose expression are known to increase to harvest nitrogen from nitrogen-containing compounds such as ammonium, nitrate, nitrite, urea, purines, pyrimidines and amino acids^38^. In D3, the upregulated genes frequently represented those involved in central carbon metabolism (CCM)^39^, which includes the pathways of TCA cycle (75% enriched), glycolysis (28.6% enriched), and the oxidative pentose phosphate pathway (OPPP) (enrichment of glucose-6-phosphate dehydrogenase activity) (Fig. S2). Moreover, mitochondrial electron transport and enzymatic activity on NADH or NADPH (85–88%) were significantly upregulated, as well as pyruvate kinase activity (62.5%) and malate dehydrogenase activity (75%), while those of photosynthesis (17%) were slightly downregulated (Fig. S2). As the cells progress to stationary phase on D5, the most obvious difference was the enrichment for multiple downregulated categories in photosynthesis: Chlorophyll biosynthetic process (75%), photosynthesis (56.5%), photosystem I (50%), photosynthesis, light harvesting (41.7%) (Fig. S3). Nevertheless, TCA cycle (37.5%) and glycolysis (14.3%) continue to be upregulated, albeit to a lesser degree. Surprisingly, FA biosynthesis (46.1%) and acetyl-CoA carboxylase activity (66.7%) were downregulated in N-replete samples, suggesting a repression of *de novo* FA synthesis. Likewise, KEGG pathway analysis reflect enrichments in carbon metabolism, TCA cycle, and pyruvate metabolism for both D3 and D5, while only D5 had significant enrichments in glycolysis and photosynthesis (Fig. S4).

### Coordinated expression of central carbon metabolism genes for storage compound synthesis

**Figure 4:**
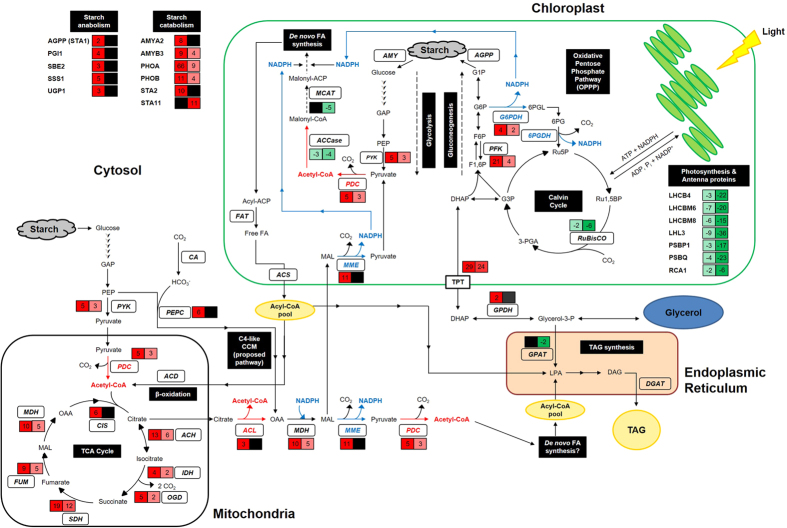
Proposed scheme of transcriptome changes to the central carbon metabolism in *D. tertiolecta* upon nitrogen depletion. Arrows represent potential carbon fluxes. Enzymes are in bold italics. Blue arrows represent reducing power (NADPH) and red arrows represent acetyl-CoA. Black boxes denote pathway names. A recently proposed C4-like carbon-concentrating mechanism (CCM) in microalgae^78^,^79^,^80^,^81^ is shown to depict an alternative route for carbon assimilation. Neutral lipid droplets found in microalgae consist mostly of triacylglycerols (TAGs), formed by combining FAs and glycerol. ***Legend:*** ACCase, acetyl-CoA carboxylase; ACD, acyl-CoA dehydrogenase; ACL, ATP-citrate lyase; ACS, acyl-CoA synthetase; AGPP, ADP-glucose pyrophosphorylase; AMY, amylase; CA, carbonic anhydrase; DGAT, diacylglycerol acyltransferase; DHAP, dihydroxyacetone phosphate; F1,6 P, fructose 1,6-bisphosphate; F6P, fructose 6-phosphate; FAT, fatty acyl-acyl carrier protein (ACP) thioesterase; G1P, glucose 1-phosphate; G6P, glucose 6-phosphate; G6PDH: G6P dehydrogenase, GAP, glyceraldehyde 3-phosphate; GPAT, glycerol-3-phosphate acyltransferase; GPDH, glycerol-3-phosphate dehydrogenase; MAL, malate; MDH, malate dehydrogenase; MME: NADP-malic enzyme; OAA, oxaloacetate; PDC, pyruvate dehydrogenase complex; PEP, phosphoenolpyruvate; PEPC, PEP carboxylase; PK, pyruvate kinase; Ru5P, ribulose 5-phosphate; Ru1,5BP, ribulose 1,5-bisphosphate; RuBisCO, Ru1,5BP carboxylase/oxygenase; TPT, triose phosphate translocator; 3-PGA, 3-phosphoglycerate; 6PGDH, 6-phosphogluconate dehydrogenase. Please refer to Supplementary Information for abbreviations of genes involved in starch synthesis and photosynthesis and antenna proteins.

Photosynthetic components and antenna proteins were severely affected in N-deplete cells, as evidenced by the global downregulation of genes encoding for light-harvesting complexes, photosystem, and RuBisCO activase, an enzyme catalyzing the activation of RuBisCO (Figs 4, S8, S9). Nonetheless, carbon continued to be assimilated as seen from the upregulation of three isoforms of gamma carbonic anhydrase (CAG1, CAG2, CAG3) in D3 (Supplementary dataset), which would provide a source of bicarbonate from CO_2_ assimilation. Pyruvate phosphate dikinase (PPD2) was upregulated by 23-fold on D3 and 4-fold on D5. PPD2 is used in the C4 carbon-concentrating mechanism pathway, where it converts pyruvate to PEP, which then reacts with bicarbonate to produce oxaloacetate (Fig. 4).

The CCM pathways were notably active during N-depletion. Glycolysis breaks down glucose to form pyruvate and acetyl-CoA. Phosphofructokinase (PFK2), pyruvate kinase (PYK1,2) and the E2 subunit of pyruvate dehydrogenase complex (DLA1; dihydrolipoamide acetyltransferase) were upregulated, forming pyruvate and acetyl-CoA which feeds into the TCA cycle. Similarly, genes in the TCA cycle were simultaneously upregulated; transcripts of citrate synthase (CIS1), aconitase (ACH1), isocitrate dehydrogenase (IDH2), oxoglutarate dehydrogenase (OGD1), succinate dehydrogenase (SDH2), fumarate hydratase (FUM1) and malate dehydrogenase (MDH3) were greatly enhanced on both D3 and D5 N-deplete samples (Figs 4, S5, S6). Interestingly, a triose phosphate/phosphoenolpyruvate translocator (TPT1) was dramatically upregulated 29- and 24-fold on D3 and D5. TPT1 could export dihydroxyacetone phosphate (DHAP) out of the chloroplast for use in glycerol synthesis as the expression of glycerol-3-phosphate dehydrogenase (GPDH) was also increased. Particularly, genes participating in starch metabolism were upregulated, including ADP-glucose pyrophosphorylase (STA1), starch synthase (SSS1) and UDP-glucose pyrophosphorylase (UGP1) and starch branching enzyme (SBE2), which together coordinate the synthesis of starch from glucose-1-phosphate (Figs 4, S7).

ATP:citrate lyase (ACLB1) and NADP-malic enzyme (MME2) were overexpressed, which would supply the cell with acetyl-CoA and NADPH respectively. In addition, glucose-6-phosphate dehydrogenase (G6PDH), a provider of reducing power from the OPPP, was also upregulated. Surprisingly, genes involved in the FA synthesis pathway were largely unchanged except for some downregulation; expression levels of malonyl-CoA-acyl carrier protein transacylase (MCAT) and acetyl-CoA carboxylase (ACCase) subunits were depressed.

### Comparing the N-depletion response in *D. tertiolecta* and other oleaginous and non-oleaginous microorganisms

**Figure 5:**
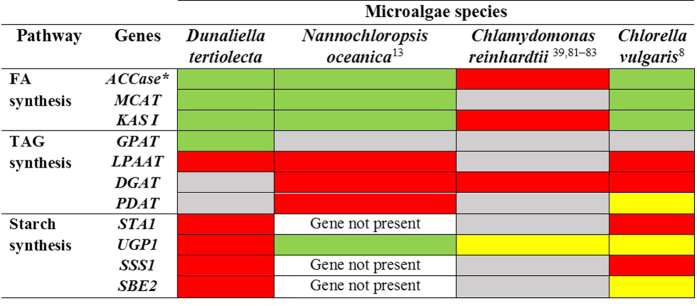
Comparison of representative genes involved in fatty acid, triacylglycerol and starch synthesis in oleaginous and non-oleaginous microalgae. Red bars: Gene expression up-regulated, Green bars: Gene expression down-regulated, Grey bars: Gene expression unchanged, Yellow bars: Gene not identified in transcriptome analysis. For more information regarding fold change, please refer to Supplementary Information Table S5. *ACCase denotes the *Acetyl-CoA biotin carboxyl carrier subunit* as it was the most reported gene among the literature.

**Figure 6:**
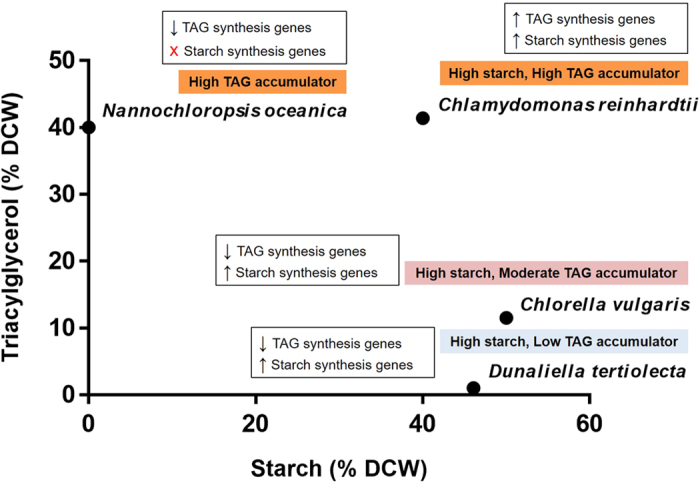
Differences in starch and TAG contents in oleaginous and non-oleaginours microalgae upon nitrogen depletion. Refer to Supplementary Information Table S4 and S5 for more information on the composition of storage compounds and gene expression in N-replete and N-deplete conditions.

To evaluate the differences in storage product accumulation between oleaginous and non-oleaginous microalgae during N-depletion, we compared the published physiological and transcriptomic data of three widely studied oleaginous species: *Nannochloropsis oceanica*^7^,^40^, *Chlamydomonas reinhardtii*^17^,^41^,^42^, and *Chlorella vulgaris*^43^ (Figs 5 and 6). *D. tertiolecta* accumulates very low amounts of TAGs in N-replete and N-deplete conditions (0.2 and 1% DCW respectively), but stores a large amount of starch, thus making it a high starch and low TAG accumulator (Fig. 6, Table S4). Interestingly, although *N. oceanica* – a marine microalga alike *D. tertiolecta* – stores the same amount of TAGs under N-replete conditions, it rapidly accumulates TAGs under N-deplete conditions (up to 40% DCW); it however lacks starch as a storage compound^13^,^44^. *C. reinhardtii* and *C. vulgaris*, both heterotrophically grown microalgae, accumulates high amounts of starch and TAGs during N-depletion.

Analysis of the expression of key genes in the FA, TAG and starch synthesis pathways showed that upon N-depletion, the oleaginous microalga *N. oceanica* and moderate TAG accumulator *C. vulgaris* experienced downregulation in the FA synthesis pathway, a pattern similar to that in the low TAG-accumulating *D. tertiolecta* from this study (Fig. 5, Table S5). In contrast, *C. reinhardtii* had moderate overexpression of its FA synthesis genes. TAG synthesis genes had variable changes in each microalga: In *D. tertiolecta* GPAT was downregulated while LPAAT was upregulated, *N. oceanica* and *C. reinhardtii* genes were mostly unchanged, while *C. vulgaris* genes were upregulated. Noticeably, *D. tertiolecta* has most of its starch synthesis genes upregulated, suggesting a coordinated push towards starch accumulation during N-depletion.

## Discussion

In microalgae, TAGs and starch are the two primary energy storage products and both synthesis pathways share common carbon precursors. It has been reported that oleaginous microalgae are able to accumulate large amounts of TAGs (up to 60% of dry weight) under N-depletions^41^,^42^,^43^,^44^,^45^,^46^. Although these studies show that N-depletion enabled oleaginous cells to channel carbon to TAGs, little attention has been paid to non-oleaginous species which prefer alternative carbon stores such as starch and glycerol, and the transcriptomic basis governing these carbon fluxes. In this study, we tracked the storage product accumulation and global gene expression profile of the halophilic, high-starch accumulating microalga *D. tertiolecta* upon N-depletion, and compared its preference of storage products with other oleaginous microalgae. A coordinated upregulation of genes involved in central carbon metabolism was identified in exponentially growing cells, suggesting that the genetic response to N-depletion occurs prior to the cessation of cell growth (i.e. stationary phase). Surprisingly, the transcriptome profile of *D. tertiolecta* – specifically the upregulation of CCM genes contributing to the supply of acetyl-CoA and NADPH – were vastly similar to oleaginous microalgae including *N. oceanica*^7^ and *C. reinhardtii*^47^. The increased transcript levels of TCA cycle genes and genes contributing to reducing power such as G6PDH and ME were correspondingly observed in heterotrophic fungi such as *Mortierella alpina*^48^ and *Mucor circinelloides*^49^,^50^ to be critical determinants of lipid synthesis.

Nitrogen is a key component for the synthesis of chlorophyll and essential proteins of the photosystem (e.g. LHCII apoprotein), thus the restriction of nitrogen supply could hinder both structural and physiological components of photosynthesis^51^. The observed drop in chlorophyll content and decreased PSII quantum yield (Fig. 1e,f) coincides with previous reports that demonstrated the degradation of chlorophyll and carotenoids in *D. tertiolecta* upon nitrogen starvation^51^,^52^,^53^. Corresponding downregulation of most light harvesting complex genes (Figs S8, S9) suggest that N-depletion triggered a response of the cell to reorganize its photosynthetic apparatus. Multiple studies of nutrient limitation in microalgae point to the degradation of thylakoid membranes in favour of the *de novo* synthesis of TAGs where intracellular membrane remodeling substantially contribute to neutral lipid accumulation^54^,^55^,^56^,^57^. Our present study shows that *D. tertiolecta* is able to accumulate up to six times more neutral lipids under N-deplete conditions (Fig. 2b), but concurrently experienced a substantial decrease in total lipid content relative to N-replete samples (9.5% of DCW in N-replete vs. 5.8% of DCW in N-deplete) (Fig. 2c). This is similar to other researchers who observe similar declines in total fatty acid content in *D. tertiolecta* under N-depletion (7.5% of DCW in N-sufficient vs 5.9% of DCW in N-deficient)^29^. The parallel increase in neutral lipids could be explained by a dramatic shift in intracellular processes, from the catabolic degradation of thylakoid membranes (which contribute to total lipids) to the anabolic reactions of storage compound accumulation (neutral lipids and starch content). This notion is supported by a recent report which showed that when cultured under N-depleted conditions, *D. tertiolecta* had significant decreases in the lipid classes of diacylglyceryltrimethylhomoserine (DGTS) and digalactosyldiacylglycerol (DGDG), a main component of chloroplast membranes^52^. This suggests the occurrence of a major remodeling of lipid membranes during nitrogen starvation in *D. tertiolecta*, a response akin to other microalgae^54^,^55^,^56^,^57^.

Glycolytic genes that shunt carbon precursors to *de novo* FA synthesis, including phosphofructokinase, pyruvate kinase and pyruvate dehydrogenase complex (PDC), are upregulated in *N. oceanica* and *C. reinhardtii* under N-depleted conditions correlating with TAG accumulation up to 40% DCW, despite a downregulation of the genes directly involved in TAG and FA synthesis^7^,^58^. This transcriptome response is unexpectedly similar to *D. tertiolecta* in this study (Fig. 4), which accumulates more starch (46% DCW) than lipids (Figs 2f and 5). Even more striking is the similarity in the overexpression of genes involved in the TCA cycle in the mitochondria (Fig. 4), which is also greatly enhanced in *N. oceanica* and was attributed to the utilization of carbon skeletons derived from membrane lipids to produce energy for TAG or its precursors^7^. As *Nannochloropsis* cells lack starch synthesis genes consistent with the absence of pyrenoid starch^13^,^59^,^60^, it may be logical the excess carbon and energy derived from the breakdown of membrane lipids are channeled to TAGs. However, in *D. tertiolecta* the response was to channel more carbon to starch instead of TAGs, as can be seen from the synchronized upregulation of genes in gluconeogenesis and starch synthesis genes (Table S5). Thus, the increased activity of CCM pathways and genes contributing to the generation of acetyl-CoA and NADPH may merely be a general response of microalgae cells to N-depletion rather than a determinant of oleaginicity. Indeed, it has been previously proposed that the CCM could play varied roles upon N-depletion; the pathways could be involved in providing carbon skeletons, for nitrogen reassimilation, or for channeling excess carbon into FA synthesis^61^. Noting that most TCA cycle enzymes are bidirectional, Sweetlove *et al.*^62^ proposed that the TCA cycle could catalyze reverse reactions and be highly adaptable to changing conditions of the cell. Hence, the TCA cycle may act as a central hub, balancing between demands for specific carbon skeletons for anabolic processes, and the energetic needs of the cell in absence of photosynthetic activity as they produce reducing equivalents for ATP synthesis.

The preference to store carbon as starch may stem from the fact that it is energetically more favourable to make than TAGs, and is the preferential reserve which is rapidly mobilized during switch from N-deplete to N-replete conditions^17^. On a per carbon basis, a 55-carbon TAG molecule requires 6.25 ATP and 2.93 NADPH, while 55 carbon units of starch requires 4.16 ATP and 2 NADPH^33^; TAG synthesis require 50% more ATP and 45% more NADPH per carbon to make. While TAGs return more ATP than sugars during metabolism, the energy recovered is less than the energy invested in its synthesis^33^,^34^, chiefly due to the carbon lost during the conversion of pyruvate to acetyl-CoA by PDC. Much of this begs the question: Why do some microalgae prefer to store more TAGs and others prefer to store predominantly starch, while some are able to store moderately equal amounts of both? One possible explanation is that as lipids require more ATP and reducing power than starch for production, photoheterotrophically grown cells such as *C. reinhardtii* and *C. vulgaris* (Table S4) can generate more substrates needed for FA synthesis due to their ability to assimilate organic carbon sources on top of inorganic carbon fixation from photosynthesis. For instance, acetate can be directly converted into acetyl-CoA from acetyl-CoA synthetase (ACS), and incorporate immediately into the TCA cycle or used in FA synthesis by *C. reinhardtii*^34^. Likewise, *Chlorella* grown in acetate medium use ACS to directly incorporate acetate into acetyl-CoA, by-passing the PDC route for acetyl-CoA production and enabling a high rate of lipid synthesis under N-depletion^63^. Furthermore, glucose-fed *Chlorella* cultures generate more reducing power in the dark than photoautotrophically cultured *Chlorella* in the light, thus the former accumulates more lipids and less starch^64^. This explanation does not extend to *Nannochloropsis* however, due to its inability to synthesize starch^13^,^59^,^60^. Rather, it stores small amounts of carbohydrates (5–17%) while channeling majority of carbon to TAGs during N-depletion.

Microalgae of the genus *Dunaliella* dominate the ranks of top carbohydrate producers, and *D. tertiolecta* – with the ability to accumulate up to 63% DCW in carbohydrates – leads the list^12^. In this study, we show that *D. tertiolecta* rapidly accumulates starch in response to N-depletion, suggesting that *D. tertiolecta* cells tend to favor starch over TAGs as an efficient strategy for chemical energy accumulation (Fig. 2a,f). As an obligate photoautotroph that cannot use dissolved organic compounds^32^,^65^, efficient energy storage and utilization is of paramount importance, especially since photosynthesis is inefficient with less than 8% conversion of solar energy chemical energy^33^. Notably, starch reserves provide the energy required in the dark for DNA replication and general cell metabolism^66^. This is in line with the increase in gene expression of starch-degrading enzymes in *D. tertiolecta* during N-depletion (Fig. 4, Fig. S7), when photosynthesis is compromised. *D. tertiolecta* also specifically degrade starch in high salinity stress conditions to yield DHAP which can be used to produce glycerol^67^, making starch a more suitable storage compound as the cells can respond to both nutrient deficiency and salinity stress, conditions which could fluctuate greatly in their natural environments^68^. In light of these observations, it is reasonable to assume that carbon would be channeled to TAG production once starch synthesis is blocked. However, this notion was refuted as there was no increase in oil among three starchless *C. reinhardtii* mutants examined^17^, suggesting that blocking starch synthesis does not result in oil accumulation. Preference of starch and TAG accumulation in microalgae may instead stem from natural evolutionary patterns and exposure to different carbon sources.

*D. tertiolecta* was previously reported to accumulate and release glycerol into the external medium, and that the process was not affected by nutrient starvation or cell death^36^. In this study, we show that under N-depletion increased amounts of glycerol were present in the extracellular medium (Fig. 2e), indicating that glycerol synthesis and export is a response to nutrient stress. This is supported by the marked upregulation of a triose phosphate translocator, which exports photosynthetically fixed carbon or carbon derived from starch breakdown from the chloroplast to the cytosol, where the enzyme GPDH converts into glycerol (Fig. 4). *D. tertiolecta* is known to uptake exogenous glycerol in response to stress^69^, and as releasing glycerol to the external medium provides the cells with an expansive carbon sink not restricted by cell size, the extracellular glycerol may serve as a readily utilizable carbon source for the cells in anticipation of improving nutrient environments^36^.

The present study identifies the unique transcriptome signatures of *D. tertiolecta*, a high starch-accumulating microalga in response to N-depletion. In particular, its coordinated upregulation of genes involved in starch synthesis could be attributed to its preference to store starch over TAGs. However, *D. tertiolecta* also shares similar upregulation of CCM genes as the oleaginous microalga *N. oceanica*, where CCM pathways were suggested to provide substrates for FA and TAG synthesis during N-depletion. The perplexing similarities and differences in transcriptomic and metabolic responses of oleaginous and non-oleaginous microalgae thus demands a relook into the role of biochemical pathways governing the allocation of carbon and energy in the cell. Addressing these issues would advance our understanding of starch and lipid synthesis in microalgae as well as facilitate the genetic engineering of microorganisms designed to accumulate either storage product of interest.

## Methods

### Microalgae Strain and Culture Conditions

The *Dunaliella tertiolecta* strain (UTEX LB 999) was obtained from the University of Texas at Austin (UTEX) and maintained in sterile ATCC-1174 DA medium under nitrogen-replete condition of 5 mM KNO_3_ (American Type Culture Collection at Manassas, Virginia) containing 0.5 M NaCl (Table S1). The cells were grown in 50-mL batch cultures on a rotary shaker at 25 °C and illuminated with 30 μmol photons m^2^/s under a photoperiod of 14 h Light/10 h Dark, with or without 5% CO_2_ aeration. Cell densities were determined using an automated cell counter (TC20^TM^ automated cell counter, Biorad Laboratories). Prior to counting, *D. tertiolecta* cells were fixed with 2% paraformaldehyde. Optical density measurements (OD_680_) were conducted with an UV spectrophotometer (Genesys 10 S UV-VIS, ThermoFisher Scientific). Biomass was determined by dry cell weight (DCW) (g/mL) measurement and combined with cell density (cells/mL) to get dcw per cell (g/cell). Ten-milliliter (10 mL) of cells were collected by filtration on pre-weighed Advantec GB-140 filter paper (0.4 μm pore size; diameter 47 mm), and washed with isotonic 0.5 M ammonium formate (40 mL) to remove salts without causing the cells to burst. Cells captured on filter paper discs were dried in oven at 95 °C until the weight was constant. Work conducted throughout the paper is based on biological triplicates unless otherwise stated.

### Nitrogen Depletion and Cultivation

*D. tertiolecta* subjected to nitrogen depletion (N-depletion) were grown in nitrogen-limited media (ATCC-1174 DA medium containing 0.5 mM KNO_3_, equivalent to 10% of original KNO_3_ concentration; K substituted with KCl) (Table S1). N-depletion was achieved by harvesting exponentially growing cells (cell density approximately 3 × 10^6^ cells/mL) and twice washing them with fresh ATCC medium containing 10% KNO_3_. All experimental cultures began at an OD_680_ of 0.1 for standardization. Nitrate concentration was determined using a UV spectrophotometric method measuring the difference between OD_220_ and OD_275_, and converted with a standard curve (Fig. S1). Cells were harvested at regular time intervals corresponding to exponential, late-exponential and stationary phases for RNA extraction, neutral lipid analysis, fluorescence microscopy, and biomass measurements. Experiments were conducted as independent biological triplicates.

### Total Organic Carbon, chlorophyll, photosynthetic yield, starch and glycerol measurements

Intracellular total organic carbon (TOC) content was measured using an automated High Temperature Combustion TOC Analyzer (LOTIX; Teledyne Tekmar), installed with a Non-Dispersive Infrared Detector. Cell densities of 3 × 10^7^ cells were harvested, spun down and washed with 1 mL of 0.08 M NaCl, reconstituted in ultrapure water to a final volume of 20 mL, and transferred to the TOC analyzer for analysis.

Starch content was measured using the Starch Assay Kit (STA20; Sigma-Aldrich), performed according to the manufacturer’s instructions. Glycerol content was measured using the Free Glycerol Determination Kit (FG0100; Sigma-Aldrich) as previously reported by Chow *et al.*^35^. Harvested cells were centrifuged (10,000 g for 10 mins, 4 °C) and the cell pellet and supernatant were separated for measurement of intracellular and extracellular glycerol respectively. For intracellular glycerol, the cell pellet was washed with equivalent volume of 0.5 M NaCl and centrifuged again to remove remaining extracellular glycerol. The cell pellet was then resuspended in 200 μL of ultrapure water, vortexed and boiled for 15 mins. Subsequently, the samples were centrifuged to remove cell debris, and the supernatant collected for intracellular glycerol analysis.

Photosynthetic yields (maximum efficiency of photosystem II; F_v_/F_m_) were evaluated using chlorophyll fluorescence measured with AquaPen-C fluorometer (AP-C 100/USB; Photon Systems Instruments). Cells were dark-adapted for 5 mins before using the fluorometer to measure F_v_/F_m_ values according to the user’s manual.

For chlorophyll measurements, cells were centrifuged (10,000 g for 10 mins, 4 °C), supernatant were removed, and the cell pellet resuspended in 0.5 mL DMSO and vortexed for 1 min until the pellet disintegrated. An equivalent volume of 90% acetone was added and mixed well, followed by centrifugation to remove cell debris. The supernatant (extraction volume of 800 μL) was used for measuring absorbances at 630 nm, 647 nm, 664 nm, 665 nm, and 750 nm using a UV spectrophotometer. Correction of pheopigments was done by adding 40 μL of 1 M HCl to To the mixture and incubating at room temperature for 90 secs. Absorbances were measured again at 665 nm and 750 nm. Chlorophyll amounts were calculated according to trichromatic equations^70^ and the monochromatic equation for pheopigment correction^71^.

1. *Trichromatic equations:*


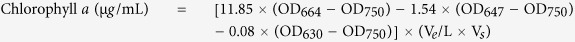



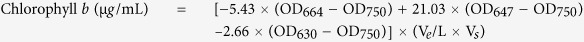


2. *Monochromatic equation for pheopigment correction:*


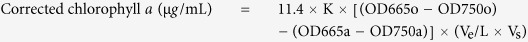


*where*


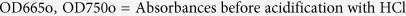



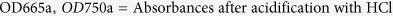


L = Light path of the cuvette (cm)

V_e_ = Extraction volume (mL)

V_s_ = Sample volume which was harvested (mL)

R=maximum absorbance ratio of OD665o/OD665a in the absence of pheopigments = 1.7 K = R/(R−1) = 2.43

Total chlorophyll (μg/mL) = Chlorophyll *b* (μg/mL) + Corrected chlorophyll *a* (μg/mL)

### Neutral Lipid Quantification

A modified Nile Red staining method^28^ was used to quantify intracellular TAGs. Briefly, cells were harvested by centrifugation (3000 *g* for 10 min at 4 °C), supernatant was removed and the pellet resuspended in fresh 0.5 M ATCC-1174 DA media to an OD_680_ of 0.3. Triolein was used as a standard for determining neutral lipid concentrations by Nile Red (Fig. S10). Two hundred microliters of triolein standards (40, 20, 10, 5, 2.5, 0 μg/mL) and cell suspensions were loaded as technical triplicates onto a 96-well black, clear bottom plate (CLS3603; Sigma-Aldrich). Prior to staining, Nile red stock is diluted in acetone to obtain a working solution (25 μg/mL), and 2 μL of the Nile red working solution is added to each well of sample and standard, followed by a 5 min incubation in the dark. Fluorescence of each sample was detected using a microplate reader (Infinite M200 PRO, Tecan) at excitation and emission wavelengths of 524 nm and 586 nm. Fluorescence imaging of Nile Red-stained cells was performed with an automated fluorescence microscope (Olympus BX63). Acquisition and processing of data was done using the cellSens software.

### Total Lipid Analysis by Gas Chromatography-Mass Spectrometry

To analyze the accumulation of total lipids, cells were harvested, snap-frozen in liquid nitrogen and stored at −80 °C until analysis. Frozen culture samples were lyophilized by freeze-drying and lipids were extracted by hexane using direct transesterification^72^ as it was reported to be a convenient and accurate method for analyzing total fatty acids^73^. Biomass quantities of between 5 and 10 mg of biomass were weighed into glass 55-mL PYREX culture tubes with polytetrafluoroethylene (PTFE)-lined phenolic caps (25 mm diameter × 150 mm height, PYREX #9826–25, Corning). To each sample, 0.2 mL of chloroform-methanol (2:1, *v*/*v*) was added and mixed by vortexing, followed by simultaneous transesterification of lipids with 0.3 mL of 1.25 M methanolic HCl and vortexed to mix. An internal standard (100 μg Methyl tridecanoate, C13-Fatty Acid Methyl Ester, C13-FAME; Cat. no. 91558, Sigma-Aldrich) was included to correct for the loss of FAME during the reaction, and to correct for subsequent incomplete extraction of hexane^74^. The culture tube was then incubated in a 50 °C waterbath overnight. After 24 hours, 1 mL of hexane was added and mixed by vortex, and incubated at room temperature for 1 hour. The upper organic phase containing FAMEs was removed using a glass pipette, filtered through a 0.22-μm PTFE syringe filter (Agilent Technologies), and collected in a 250-μL glass vial insert (Part no. 5181–1270, Agilent Technologies). FAME extracts were injected into a GC system (Model 7890B, Agilent Technologies) equipped with an Agilent 19091S-433UI column (30 m × 250 μm × 0.25 μm) interfaced with a mass spectrometric detector (Model 5977 A, Agilent Technologies). Injection volume was set at 1 μL with a 5:1 split ratio at a GC inlet temperature of 250 °C. Helium was used as the carrier gas in a fixed flow of 1 mL/min throughout. Temperature program is as follows: initial oven temperature of 70 °C held for 3 mins, ramp to 130 °C at 20 °C/min, 178 °C at 4 °C/min, 190 °C at 1 °C/min, and 290 °C at 10 °C/min. The total run time was 40 minutes. Shifting of retention times (RTs) were eliminated by comparing the RTs of each FA compound to the C13-FAME internal standard. Analysis was performed using the MassHunter WorkStation Qualitative Analysis B.07.00 software (Agilent Technologies) and compounds were identified with the NIST mass spectral library (National Institute of Standards and Technology, Data Version: NIST 14).

### Preparation of cDNA Libraries for Transcriptome-sequencing

Total RNA of N-replete and N-deplete cells on day 3 and day 5 were used for RNA-seq as they correspond to late-exponential and stationary phases of cells grown under N-deplete conditions, compared to N-replete cells which are in exponential phase. RNA integrity was assessed on an Agilent 2100 Bioanalyzer (Agilent Technologies) using the RNA 6000 Nano Kit (Cat. no. 5067–1511; Agilent Technologies). Construction of cDNA was performed with approximately 2 μg of RNA using a TruSeq Stranded Total RNA LT Sample Prep Kit (Illumina) following the manufacturer’s procedures (first and second strand cDNA synthesis, 3′ end adenylation, adapter ligation, DNA fragment enrichment of ~300 bp in length). Ribo-Zero rRNA Removal Kit for Plant (Illumina) was used to reduce ribosomal RNA amount in each sample. The quality of constructed cDNA libraries and size of DNA fragments were validated on the Bioanalyzer with Agilent DNA 1000 kit (Cat. no. 5067–1505; Agilent Technologies), before being quantified by qPCR with the KAPA Library Quantification Kit for Illumina platforms (Cat. no. KK4824; Kapa Biosystems).

### RNA-Seq and Differential Gene Expression Analysis

The cDNA libraries were normalized to 10 nM, pooled in equal volumes, and sequenced for 2 × 300-bp runs (paired-end) using Illumina MiSeq Sequencer (Illumina). A set of four cDNA libraries were sequenced per run in order to generate sufficient reads for each sample. FASTQ datasets generated from Illumina Miseq were uploaded into a Partek^®^ Flow^®^ web server (version 4.0, Partek Inc.). To ensure quality, the raw data were trimmed from both ends with the following criteria: Phred-equivalent quality score of more than 20, minimum read length of 25, quality encoding = autodetect. The filtered reads were aligned with STAR aligner (version 2.3.1j; default parameters)^75^ to an assembled *D. tertiolecta* transcriptome database previously created by our colleagues^28^. Data aligned to the transcriptome from STAR were used to test for differential expression of genes between N-replete and N-deplete samples. This was performed at transcript level with the Gene-specific analysis (GSA) approach from Partek^®^ Flow^®^ (Poisson model was selected) and normalized to RPKM (Reads Per Kilobase per Million mapped reads) values. Genes were considered to be significantly differentially expressed if their expression values had at least a fold change greater than ± 2, a false discovery rate (FDR)-corrected p value of ≤ 0.05 (Benjamini-Hochberg step-up correction), and the read coverage at either of the culture conditions were ≥ 10. The RNA-seq raw data were deposited in the Sequence Read Archive database (http://www.ncbi.nlm.nih.gov/Traces/sra/) under the accession numbers SRR4011621, SRR4011622, SRR4011623, SRR4011624, SRR4011625, SRR4011626, SRR4011627, and SRR4011628.

### Functional Annotation and Biological Interpretation of RNA-seq data

Functional annotation of cDNA reads was performed with the Partek^®^ Genomics Suite^®^ (PGS) software (version 6.6, Partek Inc.). The GSA file containing filtered genes and their associated information was imported into PGS and merged with the assembled *D. tertiolecta* transcriptome annotation file according to the name of *D. tertiolecta* contigs. The annotated data was subsequently used to perform Gene Ontology (GO) enrichment with a modified *C. reinhardtii* GO annotation file^28^ downloaded from JGI website (http://jgi.doe.gov/). Representative pathways were discovered by mapping the gene names to the *C. reinhardtii* Kyoto Encyclopedia of Genes and Genomes (KEGG) database (http://www.genome.jp/) using the pathway analysis tool^76^,^77^. Both GO enrichment and KEGG pathway analyses were conducted using the Fisher’s Exact test; the analysis was restricted to pathways with more than 2 genes, and results were filtered by enrichment p-value of less than 0.05.

## Additional Information

**How to cite this article**: Tan, K. W. M. *et al.* Nitrogen-induced metabolic changes and molecular determinants of carbon allocation in *Dunaliella tertiolecta. Sci. Rep.* **6**, 37235; doi: 10.1038/srep37235 (2016).

**Publisher’s note:** Springer Nature remains neutral with regard to jurisdictional claims in published maps and institutional affiliations.

## Supplementary Material

Supplementary Information

## References

[b1] Stephens E. *et al.* Future prospects of microalgal biofuel production systems. Trends Plant Sci. 15, 554–564 (2010). 20655798 10.1016/j.tplants.2010.06.003

[b2] Gouveia L. & Oliveira A. C. Microalgae as a raw material for biofuels production. J. Ind. Microbiol. Biotechnol. 36, 269–274 (2009). 18982369 10.1007/s10295-008-0495-6

[b3] Malcata F. X. Microalgae and biofuels: A promising partnership? Trends Biotechnol. 29, 542–549 (2011). 21724282 10.1016/j.tibtech.2011.05.005

[b4] Sayre R. Microalgae: The Potential for Carbon Capture. Bioscience 60, 722–727 (2010).

[b5] Wigmosta M. S., Coleman A. M., Skaggs R. J., Huesemann M. H. & Lane L. J. National microalgae biofuel production potential and resource demand. Water Resour. Res. 47, n/a-n/a (2011).

[b6] Cai T., Park S. Y. & Li Y. Nutrient recovery from wastewater streams by microalgae: Status and prospects. Renew. Sustain. Energy Rev. 19, 360–369 (2013).

[b7] Li J. *et al.* Choreography of Transcriptomes and Lipidomes of Nannochloropsis Reveals the Mechanisms of Oil Synthesis in Microalgae. Plant Cell 26, 1645–1665 (2014). 24692423 10.1105/tpc.113.121418 PMC4036577

[b8] Fan J. *et al.* Genomic Foundation of Starch-to-Lipid Switch in Oleaginous Chlorella spp. Plant Physiol. 169, 2444–2461 (2015). 26486592 10.1104/pp.15.01174 PMC4677908

[b9] Radakovits R., Jinkerson R. E., Darzins A. & Posewitz M. C. Genetic engineering of algae for enhanced biofuel production. Eukaryot. Cell 9, 486–501 (2010). 20139239 10.1128/EC.00364-09 PMC2863401

[b10] Yu W.-L. *et al.* Modifications of the metabolic pathways of lipid and triacylglycerol production in microalgae. Microb. Cell Fact. 10, 91 (2011). 22047615 10.1186/1475-2859-10-91 PMC3234195

[b11] Hu Q. *et al.* Microalgal triacylglycerols as feedstocks for biofuel production: perspectives and advances. Plant J. 54, 621–639 (2008). 18476868 10.1111/j.1365-313X.2008.03492.x

[b12] Slocombe S. P. *et al.* Unlocking nature’s treasure-chest: screening for oleaginous algae. Sci. Rep. 5, 9844 (2015). 26202369 10.1038/srep09844 PMC5378892

[b13] Dong H.-P. *et al.* Responses of Nannochloropsis oceanica IMET1 to Long-Term Nitrogen Starvation and Recovery. Plant Physiol. 162, 1110–1126 (2013). 23637339 10.1104/pp.113.214320 PMC3668043

[b14] Xiao Y., Zhang J., Cui J., Feng Y. & Cui Q. Metabolic profiles of Nannochloropsis oceanica IMET1 under nitrogen-deficiency stress. Bioresour. Technol. 130, 731–738 (2013). 23334034 10.1016/j.biortech.2012.11.116

[b15] Jia J. *et al.* Molecular mechanisms for photosynthetic carbon partitioning into storage neutral lipids in Nannochloropsis oceanica under nitrogen-depletion conditions. Algal Res. 7, 66–77 (2015).

[b16] Li Y., Han D., Hu G., Sommerfeld M. & Hu Q. Inhibition of starch synthesis results in overproduction of lipids in Chlamydomonas reinhardtii. Biotechnol. Bioeng. 107, 258–268 (2010). 20506159 10.1002/bit.22807

[b17] Siaut M. *et al.* Oil accumulation in the model green alga Chlamydomonas reinhardtii: characterization, variability between common laboratory strains and relationship with starch reserves. BMC Biotechnol. 11, 7 (2011). 21255402 10.1186/1472-6750-11-7 PMC3036615

[b18] Brányiková I. *et al.* Microalgae–novel highly efficient starch producers. Biotechnol. Bioeng. 108, 766–776 (2011). 21404251 10.1002/bit.23016

[b19] Dragone G., Fernandes B. D., Abreu A. P., Vicente A. A. & Teixeira J. A. Nutrient limitation as a strategy for increasing starch accumulation in microalgae. Appl. Energy 88, 3331–3335 (2011).

[b20] Bumbak F., Cook S., Zachleder V., Hauser S. & Kovar K. Best practices in heterotrophic high-cell-density microalgal processes: achievements, potential and possible imitations. Appl. Microbiol. Biotechnol. 91, 31–46 (2011). 21567179 10.1007/s00253-011-3311-6 PMC3114082

[b21] Heifetz P. B., Förster B., Osmond C. B., Giles L. J. & Boynton J. E. Effects of acetate on facultative autotrophy in Chlamydomonas reinhardtii assessed by photosynthetic measurements and stable isotope analyses. Plant Physiol. 122, 1439–1445 (2000). 10759539 10.1104/pp.122.4.1439 PMC58978

[b22] Valenzuela J. *et al.* Potential role of multiple carbon fixation pathways during lipid accumulation in Phaeodactylum tricornutum. Biotechnol. Biofuels 5, 40 (2012). 22672912 10.1186/1754-6834-5-40 PMC3457861

[b23] Tang H. *et al.* Potential of microalgae oil from Dunaliella tertiolecta as a feedstock for biodiesel. Appl. Energy 88, 3324–3330 (2011).

[b24] Bouchard J. N., García-Gómez C. & Rosario Lorenzo M. & Segovia, M. Differential effect of ultraviolet exposure (UVR) in the stress response of the Dinophycea Gymnodinium sp. and the Chlorophyta Dunaliella tertiolecta: mortality versus survival. Mar. Biol. 160, 2547–2560 (2013).

[b25] Georgianna D. R. *et al.* Production of recombinant enzymes in the marine alga Dunaliella tertiolecta. Algal Res. 2, 2–9 (2013).

[b26] de Boer K., Moheimani N. R., Borowitzka M. A. & Bahri P. A. Extraction and conversion pathways for microalgae to biodiesel: a review focused on energy consumption. J. Appl. Phycol. 24, 1681–1698 (2012).

[b27] Rismani-Yazdi H., Haznedaroglu B. Z., Bibby K. & Peccia J. Transcriptome sequencing and annotation of the microalgae Dunaliella tertiolecta: pathway description and gene discovery for production of next-generation biofuels. BMC Genomics 12, 148 (2011). 21401935 10.1186/1471-2164-12-148 PMC3061936

[b28] Yao L. *et al.* RNA-Seq transcriptomic analysis with Bag2D software identifies key pathways enhancing lipid yield in a high lipid-producing mutant of the non-model green alga Dunaliella tertiolecta. Biotechnol. Biofuels 8, 191 (2015). 26613001 10.1186/s13068-015-0382-0 PMC4660794

[b29] Shin H. *et al.* Elucidation of the growth delimitation of Dunaliella tertiolecta under nitrogen stress by integrating transcriptome and peptidome analysis. Bioresour. Technol. 194, 57–66 (2015). 26185926 10.1016/j.biortech.2015.07.002

[b30] Shang C. *et al.* Discovery of genes for production of biofuels through transcriptome sequencing of Dunaliella parva. Algal Res. 13, 318–326 (2016).

[b31] Nikookar K., Moradshahi A. & Hosseini L. Physiological responses of Dunaliella salina and Dunaliella tertiolecta to copper toxicity. Biomol. Eng. 22, 141–146 (2005). 16103009 10.1016/j.bioeng.2005.07.001

[b32] Hosseini Tafreshi A. & Shariati M. *Dunaliella* biotechnology: methods and applications. J. Appl. Microbiol. 107, 14–35 (2009). 19245408 10.1111/j.1365-2672.2009.04153.x

[b33] Subramanian S., Barry A. N., Pieris S. & Sayre R. T. Comparative energetics and kinetics of autotrophic lipid and starch metabolism in chlorophytic microalgae: implications for biomass and biofuel production. Biotechnol. Biofuels 6, 150 (2013). 24139286 10.1186/1754-6834-6-150 PMC4015678

[b34] Johnson X. & Alric J. Central carbon metabolism and electron transport in Chlamydomonas reinhardtii: metabolic constraints for carbon partitioning between oil and starch. Eukaryot. Cell 12, 776–793 (2013). 23543671 10.1128/EC.00318-12 PMC3675994

[b35] Chow Y. Y. S. *et al.* Continual production of glycerol from carbon dioxide by Dunaliella tertiolecta. Bioresour. Technol. 136, 550–555 (2013). 23567730 10.1016/j.biortech.2013.03.040

[b36] Chow Y., Tu W. Y., Wang D., Ng D. H. P. & Lee Y. K. The role of micronutrients and strategies for optimized continual glycerol production from carbon dioxide by Dunaliella tertiolecta. Biotechnol. Bioeng. 112, 2163–2171 (2015). 25855006 10.1002/bit.25608

[b37] Sanz-Luque E. *et al.* THB1, a truncated hemoglobin, modulates nitric oxide levels and nitrate reductase activity. Plant J. 81, 467–479 (2015). 25494936 10.1111/tpj.12744

[b38] Park J.-J. *et al.* The response of Chlamydomonas reinhardtii to nitrogen deprivation: a systems biology analysis. Plant J. 81, 611–624 (2015). 25515814 10.1111/tpj.12747

[b39] Noor E. *et al.* Central carbon metabolism as a minimal biochemical walk between precursors for biomass and energy. Mol. Cell 39, 809–820 (2010). 20832731 10.1016/j.molcel.2010.08.031

[b40] Goncalves E. C. *et al.* Nitrogen starvation-induced accumulation of triacylglycerol in the green algae: evidence for a role for ROC40, a transcription factor involved in circadian rhythm. Plant J. 85, 743–757 (2016). 26920093 10.1111/tpj.13144

[b41] Cakmak T. *et al.* Differential effects of nitrogen and sulfur deprivation on growth and biodiesel feedstock production of Chlamydomonas reinhardtii. Biotechnol. Bioeng. 109, 1947–1957 (2012). 22383222 10.1002/bit.24474

[b42] Davey M. P. *et al.* Triacylglyceride production and autophagous responses in Chlamydomonas reinhardtii depend on resource allocation and carbon source. Eukaryot. Cell 13, 392–400 (2014). 24413660 10.1128/EC.00178-13 PMC3957581

[b43] Ikaran Z., Suárez-Alvarez S., Urreta I. & Castañón S. The effect of nitrogen limitation on the physiology and metabolism of chlorella vulgaris var L3. Algal Res. 10, 134–144 (2015).

[b44] Vieler A. *et al.* Genome, Functional Gene Annotation, and Nuclear Transformation of the Heterokont Oleaginous Alga Nannochloropsis oceanica CCMP1779. PLoS Genet. 8, e1003064 (2012). 23166516 10.1371/journal.pgen.1003064 PMC3499364

[b45] Zhu S. *et al.* Metabolic changes of starch and lipid triggered by nitrogen starvation in the microalga Chlorella zofingiensis. Bioresour. Technol. 152, 292–298 (2014). 24308944 10.1016/j.biortech.2013.10.092

[b46] Rodolfi L. *et al.* Microalgae for oil: strain selection, induction of lipid synthesis and outdoor mass cultivation in a low-cost photobioreactor. Biotechnol. Bioeng. 102, 100–112 (2009). 18683258 10.1002/bit.22033

[b47] López García de, Lomana A. *et al.* Transcriptional program for nitrogen starvation-induced lipid accumulation in Chlamydomonas reinhardtii. Biotechnol. Biofuels 8, 207 (2015). 26633994 10.1186/s13068-015-0391-z PMC4667458

[b48] Chen H. *et al.* Identification of a critical determinant that enables efficient fatty acid synthesis in oleaginous fungi. Sci. Rep. 5, 11247 (2015). 26059272 10.1038/srep11247 PMC4462047

[b49] Zhang Y., Adams I. P. & Ratledge C. Malic enzyme: the controlling activity for lipid production? Overexpression of malic enzyme in Mucor circinelloides leads to a 2.5-fold increase in lipid accumulation. Microbiology 153, 2013–2025 (2007). 17600047 10.1099/mic.0.2006/002683-0

[b50] Li Z. *et al.* Overexpression of malic enzyme (ME) of Mucor circinelloides improved lipid accumulation in engineered Rhodotorula glutinis. Appl. Microbiol. Biotechnol. 97, 4927–4936 (2013). 23179623 10.1007/s00253-012-4571-5

[b51] Young E. B. & Beardall J. Photosynthetic Function in Dunaliella Tertiolecta (Chlorophyta) During A Nitrogen Starvation and Recovery Cycle. J. Phycol. 39, 897–905 (2003).

[b52] Kim S.-H. *et al.* Effects of light intensity and nitrogen starvation on glycerolipid, glycerophospholipid, and carotenoid composition in Dunaliella tertiolecta culture. PLoS One 8, e72415 (2013). 24039760 10.1371/journal.pone.0072415 PMC3764108

[b53] Geider R., Macintyre, Graziano L. & McKay R. M. Responses of the photosynthetic apparatus of *Dunaliella tertiolecta* (Chlorophyceae) to nitrogen and phosphorus limitation. Eur. J. Phycol. 33, 315–332 (1998).

[b54] Yang Z.-K. *et al.* Molecular and cellular mechanisms of neutral lipid accumulation in diatom following nitrogen deprivation. Biotechnol. Biofuels 6, 67 (2013). 23642220 10.1186/1754-6834-6-67 PMC3662598

[b55] Simionato D. *et al.* The response of Nannochloropsis gaditana to nitrogen starvation includes de novo biosynthesis of triacylglycerols, a decrease of chloroplast galactolipids, and reorganization of the photosynthetic apparatus. Eukaryot. Cell 12, 665–676 (2013). 23457191 10.1128/EC.00363-12 PMC3647774

[b56] Urzica E. I. *et al.* Remodeling of membrane lipids in iron-starved Chlamydomonas. J. Biol. Chem. 288, 30246–30258 (2013). 23983122 10.1074/jbc.M113.490425 PMC3798491

[b57] Martin G. J. O. *et al.* Lipid profile remodeling in response to nitrogen deprivation in the microalgae Chlorella sp. (Trebouxiophyceae) and Nannochloropsis sp. (Eustigmatophyceae). PLoS One 9, e103389 (2014). 25171084 10.1371/journal.pone.0103389 PMC4149361

[b58] Shtaida N., Khozin-Goldberg I. & Boussiba S. The role of pyruvate hub enzymes in supplying carbon precursors for fatty acid synthesis in photosynthetic microalgae. Photosynth. Res. 125, 407–422 (2015). 25846135 10.1007/s11120-015-0136-7

[b59] Scholz M. J. *et al.* Ultrastructure and composition of the Nannochloropsis gaditana cell wall. Eukaryot. Cell 13, 1450–1464 (2014). 25239976 10.1128/EC.00183-14 PMC4248700

[b60] Vieler A. *et al.* Genome, functional gene annotation, and nuclear transformation of the heterokont oleaginous alga Nannochloropsis oceanica CCMP1779. PLoS Genet. 8, e1003064 (2012). 23166516 10.1371/journal.pgen.1003064 PMC3499364

[b61] Hockin N. L., Mock T., Mulholland F., Kopriva S. & Malin G. The response of diatom central carbon metabolism to nitrogen starvation is different from that of green algae and higher plants. Plant Physiol. 158, 299–312 (2012). 22065419 10.1104/pp.111.184333 PMC3252072

[b62] Sweetlove L. J. *et al.* Not just a circle: flux modes in the plant TCA cycle. Trends Plant Sci. 15, 462–470 (2010). 20554469 10.1016/j.tplants.2010.05.006

[b63] Avidan O. & Pick U. Acetyl-CoA synthetase is activated as part of the PDH-bypass in the oleaginous green alga Chlorella desiccata. J. Exp. Bot. 66, 7287–7298 (2015). 26357883 10.1093/jxb/erv424 PMC4765794

[b64] Chen T. *et al.* Light attenuates lipid accumulation while enhancing cell proliferation and starch synthesis in the glucose-fed oleaginous microalga Chlorella zofingiensis. Sci. Rep. 5, 14936 (2015). 26442783 10.1038/srep14936 PMC4595723

[b65] Segovia M., Haramaty L., Berges J. A. & Falkowski P. G. Cell death in the unicellular chlorophyte Dunaliella tertiolecta. A hypothesis on the evolution of apoptosis in higher plants and metazoans. Plant Physiol. 132, 99–105 (2003). 12746516 10.1104/pp.102.017129 PMC166956

[b66] Bišová K. & Zachleder V. Cell-cycle regulation in green algae dividing by multiple fission. J. Exp. Bot. 65, 2585–2602 (2014). 24441762 10.1093/jxb/ert466

[b67] Liska A. J., Shevchenko A., Pick U. & Katz A. Enhanced photosynthesis and redox energy production contribute to salinity tolerance in Dunaliella as revealed by homology-based proteomics. Plant Physiol. 136, 2806–2817 (2004). 15333751 10.1104/pp.104.039438 PMC523343

[b68] Oren A. The ecology of Dunaliella in high-salt environments. J. Biol. Res. (Thessalonikē, Greece) 21, 23 (2014). 10.1186/s40709-014-0023-y PMC4389652 25984505

[b69] Lin H., Fang L., Low C. S., Chow Y. & Lee Y. K. Occurrence of glycerol uptake in *Dunaliella tertiolecta* under hyperosmotic stress. FEBS J. 280, 1064–1072 (2013). 23279806 10.1111/febs.12100

[b70] Jeffrey S. W. & Humphrey G. F. New spectrophotometric equations for determining chlorophylls a, b, c1 and c2 in higher plants, algae and natural phytoplankton. Biochem Physiol Pflanz BPP (1975).

[b71] LORENZEN C. J. Determination Of Chlorophyll and Pheo-Pigments: Spectrophotometric Equations1. Limnol. Oceanogr. 12, 343–346 (1967).

[b72] Lee S. Y. *et al.* Fatty acids and global metabolites profiling of Dunaliella tertiolecta by shifting culture conditions to nitrate deficiency and high light at different growth phases. Process Biochem. 49, 996–1004 (2014).

[b73] Cavonius L. R., Carlsson N. G. & Undeland I. Quantification of total fatty acids in microalgae: Comparison of extraction and transesterification methods. Anal. Bioanal. Chem. 406, 7313–7322 (2014). 25224639 10.1007/s00216-014-8155-3 PMC4206773

[b74] Laurens L. M. L., Quinn M., Van Wychen S., Templeton D. W. & Wolfrum E. J. Accurate and reliable quantification of total microalgal fuel potential as fatty acid methyl esters by *in situ* transesterification. Anal. Bioanal. Chem. 403, 167–178 (2012). 22349344 10.1007/s00216-012-5814-0 PMC3309134

[b75] Dobin A. *et al.* STAR: Ultrafast universal RNA-seq aligner. Bioinformatics 29, 15–21 (2013). 23104886 10.1093/bioinformatics/bts635 PMC3530905

[b76] Kanehisa M. & Goto S. KEGG: kyoto encyclopedia of genes and genomes. Nucleic Acids Res. 28, 27–30 (2000). 10592173 10.1093/nar/28.1.27 PMC102409

[b77] Kanehisa M., Sato Y., Kawashima M., Furumichi M. & Tanabe M. KEGG as a reference resource for gene and protein annotation. Nucleic Acids Res. 44, D457–D462 (2016). 26476454 10.1093/nar/gkv1070 PMC4702792

[b78] Radakovits R. *et al.* Draft genome sequence and genetic transformation of the oleaginous alga Nannochloropis gaditana. Nat. Commun. 3, 686 (2012). 22353717 10.1038/ncomms1688 PMC3293424

[b79] Valledor L., Furuhashi T., Recuenco-Muñoz L., Wienkoop S. & Weckwerth W. System-level network analysis of nitrogen starvation and recovery in Chlamydomonas reinhardtii reveals potential new targets for increased lipid accumulation. Biotechnol. Biofuels 7, 171 (2014). 25663847 10.1186/s13068-014-0171-1 PMC4320484

[b80] Gargouri M. *et al.* Identification of regulatory network hubs that control lipid metabolism in Chlamydomonas reinhardtii. J. Exp. Bot. 66, 4551–4566 (2015). 26022256 10.1093/jxb/erv217 PMC4507760

[b81] Sato A., Matsumura R., Hoshino N., Tsuzuki M. & Sato N. Responsibility of regulatory gene expression and repressed protein synthesis for triacylglycerol accumulation on sulfur-starvation in Chlamydomonas reinhardtii. Front. Plant Sci. 5, 444 (2014). 25309550 10.3389/fpls.2014.00444 PMC4160968

