# Supplementary material for: Nitrogen-induced metabolic changes and molecular determinants of carbon allocation in Dunaliella tertiolecta
Source: Sci Rep. 2016 Nov 16;6:37235. doi: 10.1038/srep37235 (PMC5110973; doi:10.1038/srep37235)
Supplement: Supplementary Information [file srep37235-s1.pdf]

Nitrogen-induced metabolic changes and molecular determinants of carbon allocation in *Dunaliella tertiolecta*

Kenneth Wei Min Tan, Huixin Lin, Hui Shen, and Yuan Kun Lee

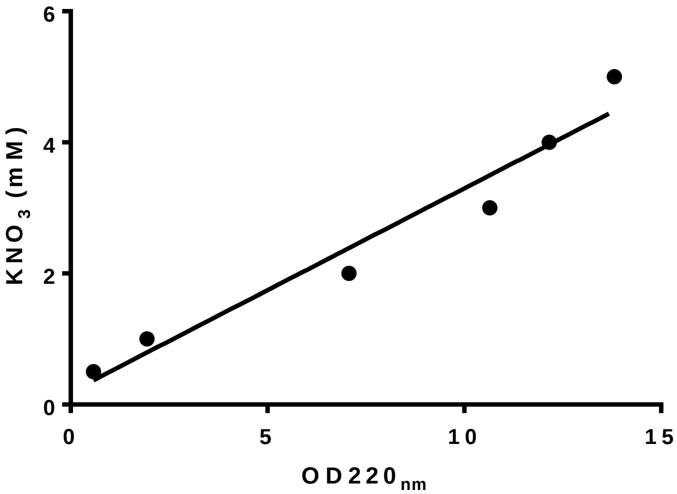

Figure S1. Standard curve for nitrate concentration in ATCC media-1174 DA media. Media samples collected in a quartz cuvette were measured at OD_{220nm} and OD_{275nm} with a UV spectrophotometer. The values of OD_{275nm} were subtracted from OD_{220nm} to correct the value.

Table S1. Media composition for ATCC-1174 DA medium. Compounds in bold are variable depending on type of media used for N-depletion experiments.

Compound	Quantity	Remarks
NaCl	29.22 g	For 0.5M NaCl.
Tris-HCl (pH 7.5)	6.024 g	
$NaHCO_3$	1.68 g	
**KNO_3**	0.505 g	For 10% KNO_3 media, KNO_3 was reduced to 0.0505 g (50.5 µg)
$MgSO_4.7H_2O$	1.232 g	
$CaCl_2$	0.033 g	
KH_2PO_4	0.014 g	
$FeCl_3$	50.0 mL	$FeCl_3$ (0.32 mg) + EDTA (5.84 mg) + Distilled Water (50 mL)
H_3BO_3	6.0 mg	
$MnCl_2.4H_2O$	99.0 µg	
$ZnCl_2$	14.0 µg	
$CoCl_2.6H_2O$	4.76 µg	
$CuCl_2.2H_2O$	34.0 ng	
Distilled water	1.0 L	
**KCl**		For 10% KNO_3 (N depletion) media only. KNO_3 was substituted with KCl for N depletion..

Table S2. Transcriptome assembly details of *D. tertiolecta* samples.

	**Day 3**	**Day 5**
**Number of genes annotated** (Total)	23,778	19,173
**Number of significantly expressed genes** (FDR-corrected p-value ≤ 0.05)	3,962	1,234
**Number of genes over/under-expressed** (≥2 or ≤ -2)	3,799	1,096
**No. of significant GO enrichment categories** (FDR-corrected p-value ≤ 0.05)	57	31
**No. of significant KEGG pathway categories** (FDR-corrected p-value ≤ 0.05)	11	17

Table S3. List of the top 10 most upregulated or downregulated genes in N-depleted *D. tertiolecta* cells.

Day	KEGG ID	Gene/Protein name	Fold change	GO/KEGG Function
**3**	CHLREDRAFT_127387	COX19 (cytochrome c oxidase assembly protein)	58,363.7	
**3**	CHLREDRAFT_152591	HMOX2 (heme oxygenase)	52,488.1	Porphyrin and chlorophyll metabolism
**3**	CHLREDRAFT_154732	Adenine nucleotide alpha hydrolases-like superfamily protein	-46,367.6	
**3**	CHLREDRAFT_182662	RPB11 (DNA-directed RNA polymerase II)	-32,666.6	Transcription of DNA to mRNA
**3**	CHLREDRAFT_162635	FAP109 (Flagellar associated protein)	36,278.6	
**3**	CHLREDRAFT_107200	RING/U-box superfamily protein	32,693.9	Ubiquitin protein ligase activity
**3**	CHLREDRAFT_192192	hypothetical protein	31,387.3	
**3**	CHLREDRAFT_116571	CPA2 (N-carbamoylputrescine amidase)	29,614.5	Arginine and proline metabolism
**3**	CHLREDRAFT_191334	FAP114 (Flagellar associated protein)	28,521.0	
**3**	CHLREDRAFT_17065	ANK14	23,938.9	
**5**	CHLREDRAFT_140618	GAP1a (glyceraldehyde 3-phosphate dehydrogenase)	1,284.0	Glycolysis / Gluconeogenesis
**5**	CHLREDRAFT_184156	FOX1 (multicopper ferroxidase)	-561.8	Porphyrin and chlorophyll metabolism
**5**	CHLREDRAFT_81856	THB1 (Putative truncated hemoglobin)	-298.4	Regulates nitrogen assimilation pathway
**5**	CHLREDRAFT_196484	PHO1 (alkaline phosphatase)	-150.3	Protein dephosphorylation
**5**	CHLREDRAFT_170417	DUR3C (urea active transporter)	72.6	urea transmembrane transporter activity
**5**	CHLREDRAFT_196479	DUR3A (urea active transporter)	65.0	urea transmembrane transporter activity
**5**	CHLREDRAFT_154212	DUR3B (urea active transporter)	52.0	urea transmembrane transporter activity
**5**	CHLREDRAFT_187840	hypothetical protein	-43.3	
**5**	CHLREDRAFT_195162	Light-harvesting complex II chlorophyll a-b binding protein M3	-43.2	Photosynthesis - antenna proteins
**5**	CHLREDRAFT_185309	Lhc-like protein Lhl3	-36.1	

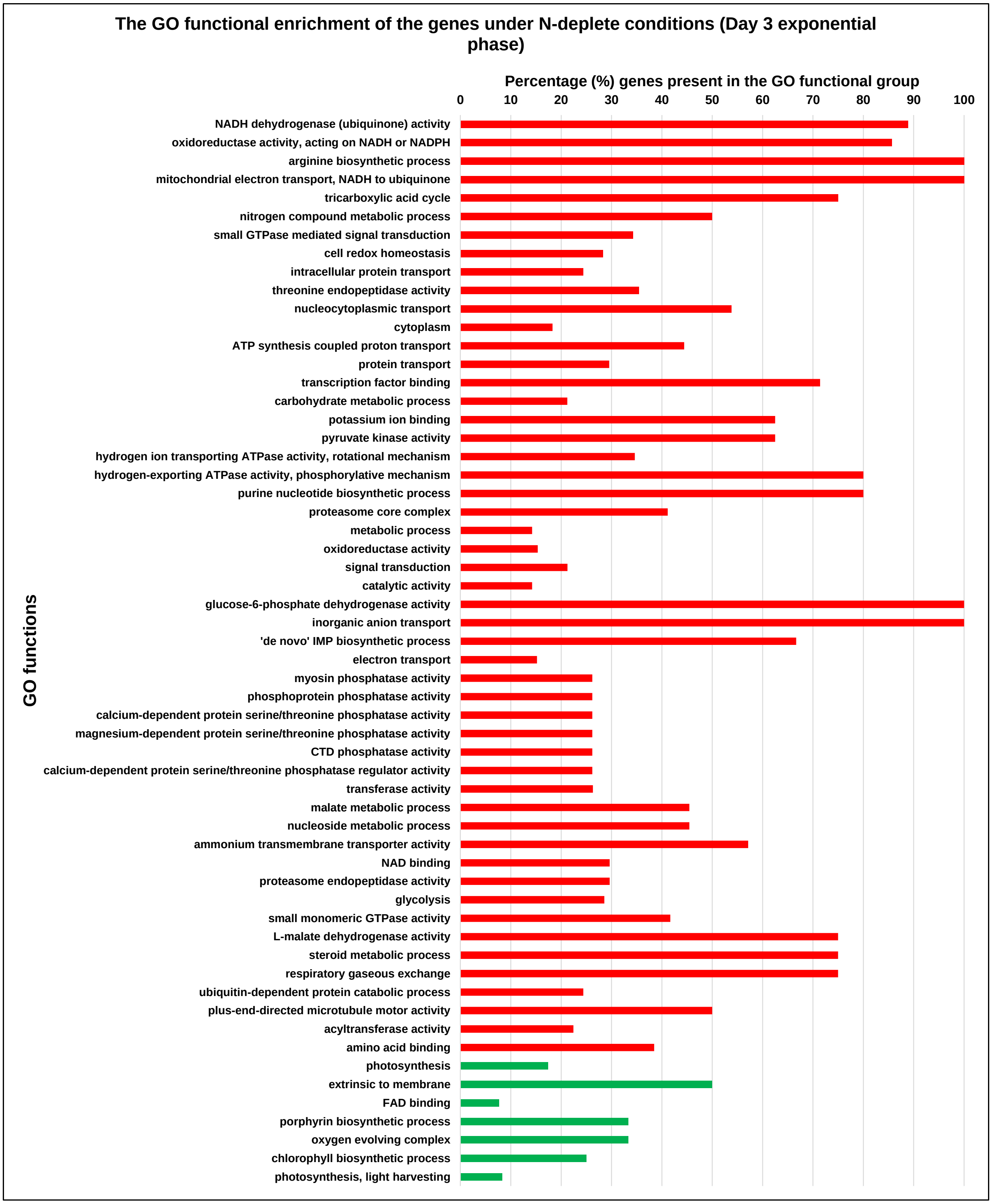

Figure S2. **GO functional enrichment of up-regulated and down-regulated genes under N-deplete conditions on Day 3 exponential phase.** Red and green bars indicate up- (≥ 2) and down- (≤ -2) regulation respectively. All data were filtered according to a FDR-corrected p value of ≤ 0.05.

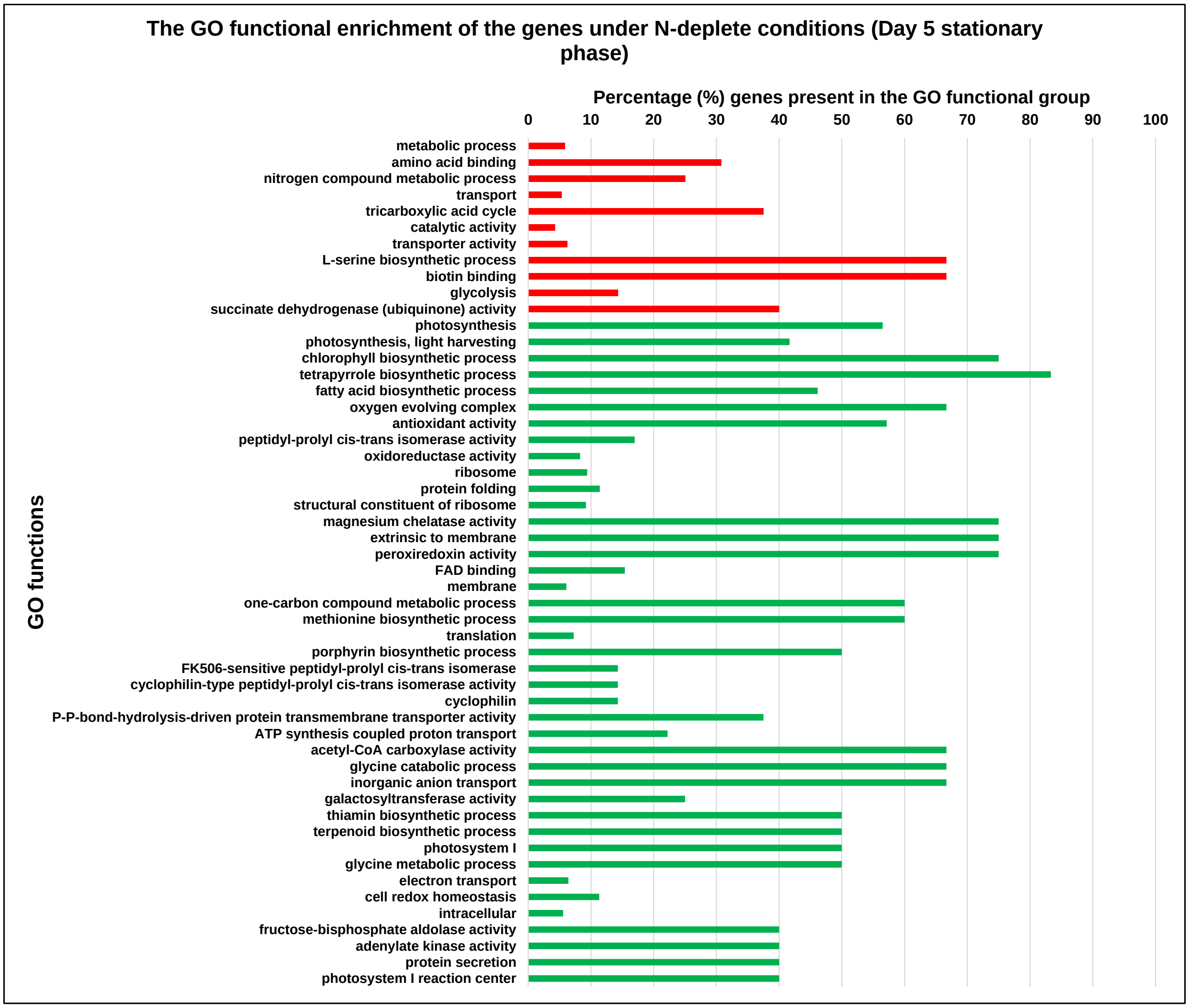

Figure S3. GO functional enrichment of up-regulated and down-regulated genes under N-deplete conditions on Day 5 stationary phase. Red and green bars indicate up- (≥ 2) and down- (≤ -2) regulation respectively. All data were filtered according to a FDR-corrected p value of ≤ 0.05.

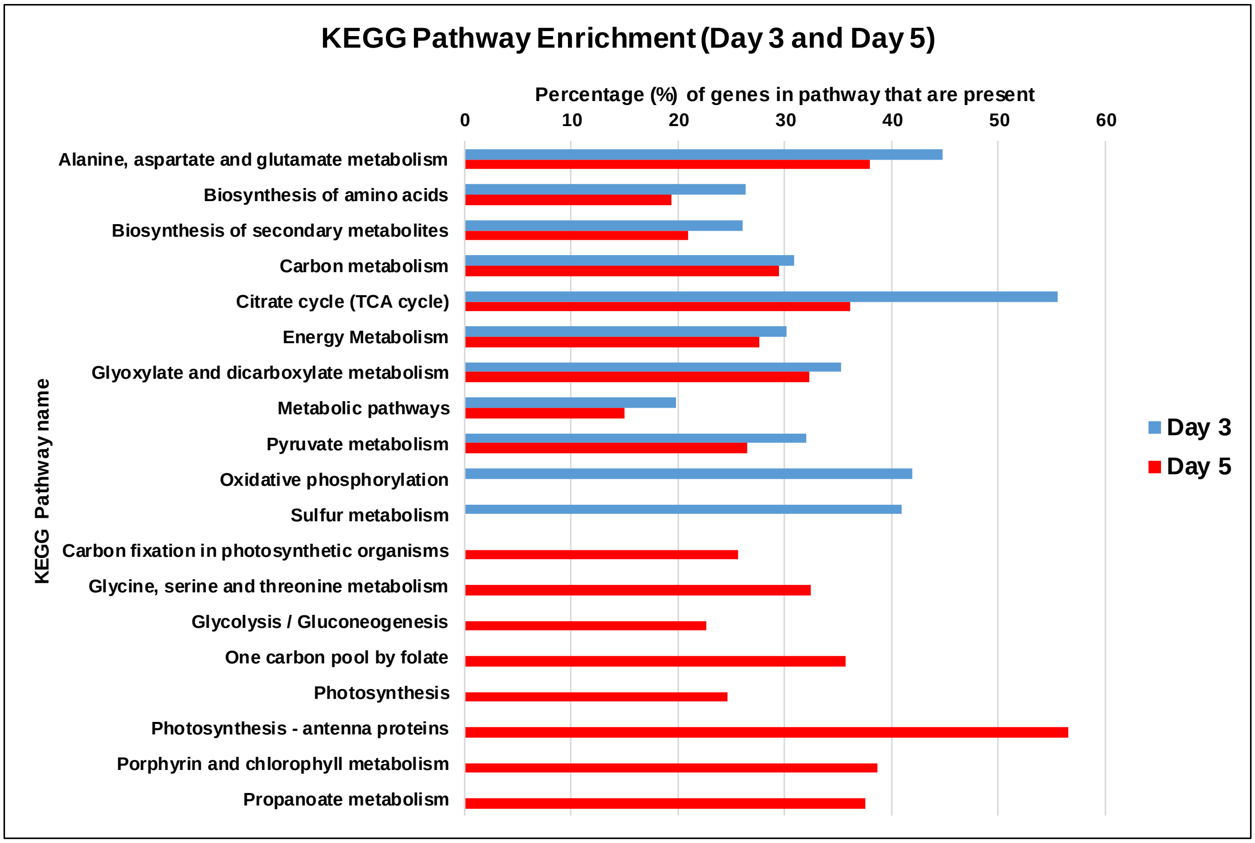

Figure S4. **KEGG pathway enrichment of genes under N-deplete conditions on Day 3 (exponential phase) and Day (5 stationary phase).** Blue bars represent the Day 3 samples and red bars represent the Day 5 samples. All data were filtered according to a FDR-corrected p value of ≤ 0.05.

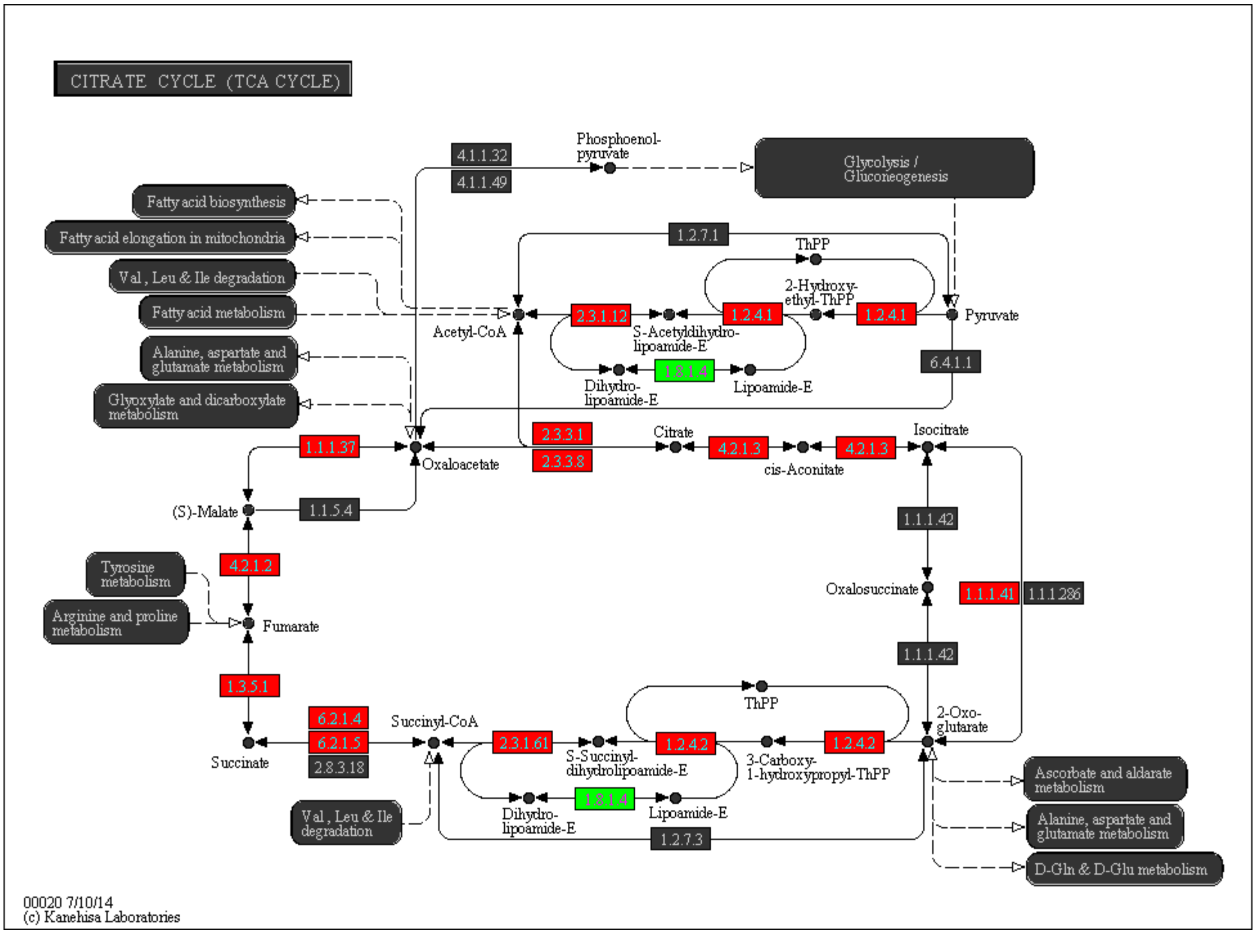

Figure S5. **KEGG pathway analysis of TCA cycle (Day 3 exponential phase).** Red and green highlighted bars indicate up- (≥ 2) and down- (≤ -2) regulation respectively. EC 2.3.1.12: PDH E2 component (Dihydrolipoamide acetyltransferase), EC 1.2.4.1: PDH2 (Pyruvate dehydrogenase E1 beta subunit), EC 1.8.1.4: PDH (Dihydrolipoamide dehydrogenase), EC 2.3.3.1/8: ACLB1 (ATP citrate lyase, subunit B), EC 4.2.1.3: ACH (Aconitase), EC 1.1.1.41: IDH (Isocitrate dehydrogenase), EC 1.2.4.2: OGD (Oxoglutarate dehydrogenase), EC 2.3.1.61: OGD2 (Dihydrolipoamide succinyltransferase), EC 6.2.1.4: SCLA (Succinyl coenzyme A synthetase subunit A), EC 6.2.1.5: SCLB (Succinyl coenzyme A synthetase subunit B), EC 1.3.5.1: SDH (Succinate dehydrogenase), EC 4.2.1.2: FUM (Fumarate hydratase), EC 1.1.1.37: MDH (Malate dehydrogenase). Images are obtained by KEGG (Kyoto Encyclopedia of Genes and Genomes).

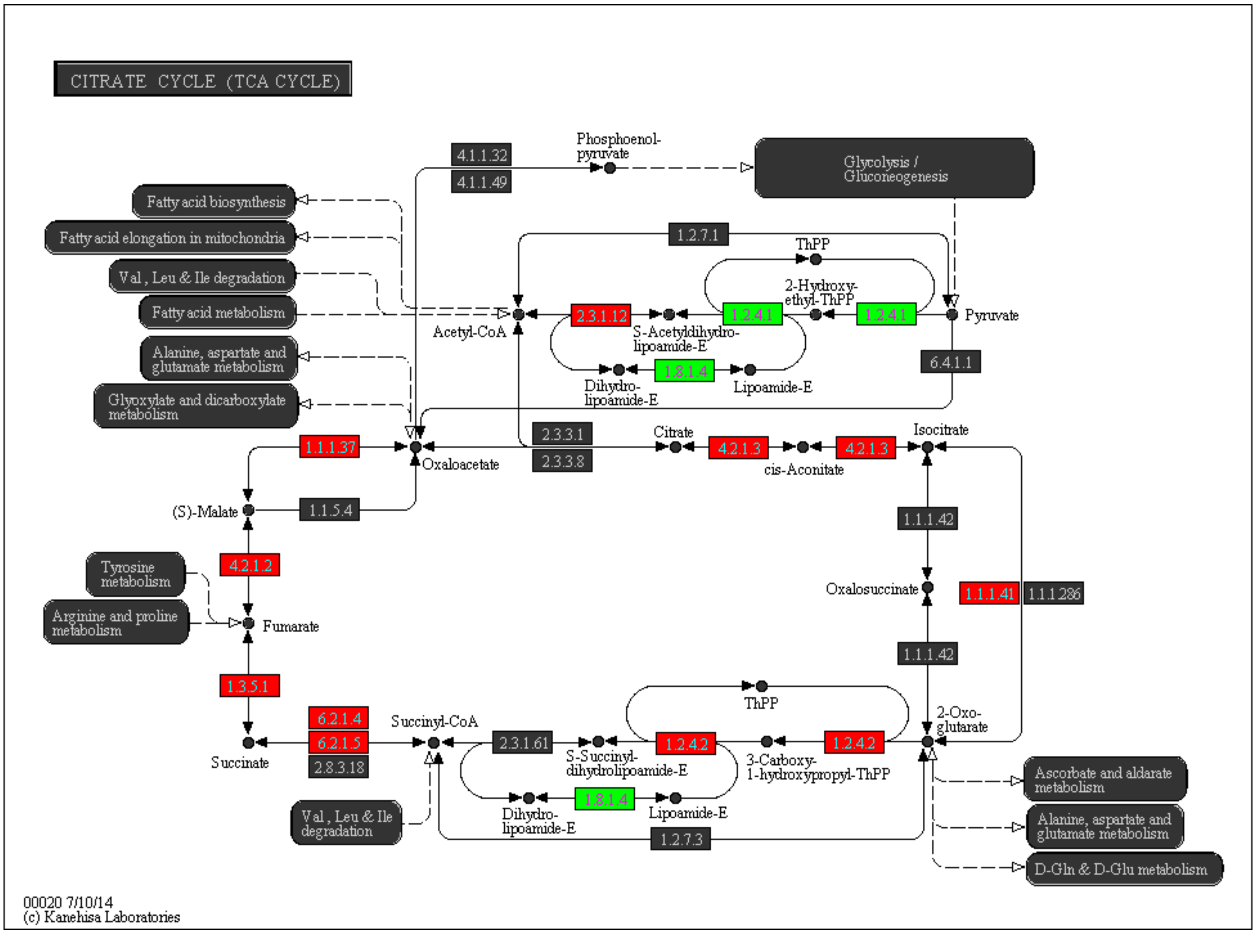

Figure S6. **KEGG pathway analysis of TCA cycle (Day 5 stationary phase).** Red and green highlighted bars indicate up- (≥ 2) and down- (≤ -2) regulation respectively. EC 2.3.1.12: PDH E2 component (Dihydrolipoamide acetyltransferase), EC 1.2.4.1: PDH2 (Pyruvate dehydrogenase E1 beta subunit), EC 1.8.1.4: PDH (Dihydrolipoamide dehydrogenase), EC 2.3.3.1/8: ACLB1 (ATP citrate lyase, subunit B), EC 4.2.1.3: ACH (Aconitase), EC 1.1.1.41: IDH (Isocitrate dehydrogenase), EC 1.2.4.2: OGD (Oxoglutarate dehydrogenase), EC 2.3.1.61: OGD2 (Dihydrolipoamide succinyltransferase), EC 6.2.1.4: SCLA (Succinyl coenzyme A synthetase subunit A), EC 6.2.1.5: SCLB (Succinyl coenzyme A synthetase subunit B), EC 1.3.5.1: SDH (Succinate dehydrogenase), EC 4.2.1.2: FUM (Fumarate hydratase), EC 1.1.1.37: MDH (Malate dehydrogenase). Images are obtained by KEGG (Kyoto Encyclopedia of Genes and Genomes).

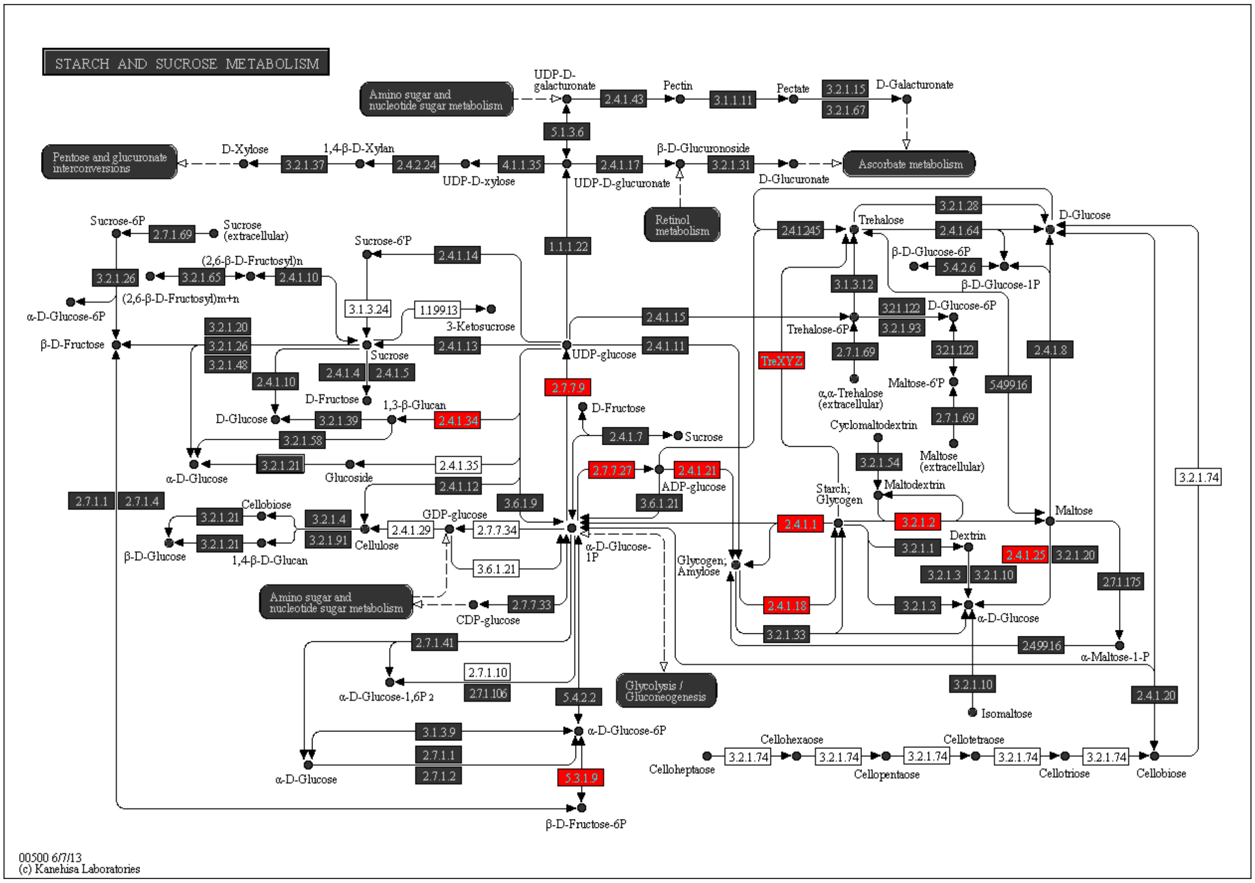

Figure S7. **KEGG pathway analysis of Starch and sucrose metabolism (Day 3 exponential phase).** Red highlighted bars indicate up- (≥ 2) and down- (≤ -2) regulation respectively. EC 2.4.1.34: STA2 (granule-bound starch synthase I; 1,3-beta-glucan synthase), EC 5.3.1.9: PGI1 (glucose-6-phosphate isomerase), EC 2.7.7.9: UGP1 (UDP-glucose pyrophosphorylase; UTP—glucose-1-phosphate uridylyltransferase), EC 2.7.7.27: STA1/AGPP (Glucose-1-phosphate adenylyltransferase; ADP-glucose pyrophosphorylase), EC 2.4.1.21: SSS (Starch synthase), EC 2.4.1.18: SBE (Starch branching enzyme), EC 2.4.1.1: PHOA/B (Starch phosphorylase), EC 3.2.1.2: AMYB3 (Beta-amylase), EC 2.4.1.25: STA11 (4-alpha-glucanotransferase). Images are obtained by KEGG (Kyoto Encyclopedia of Genes and Genomes).

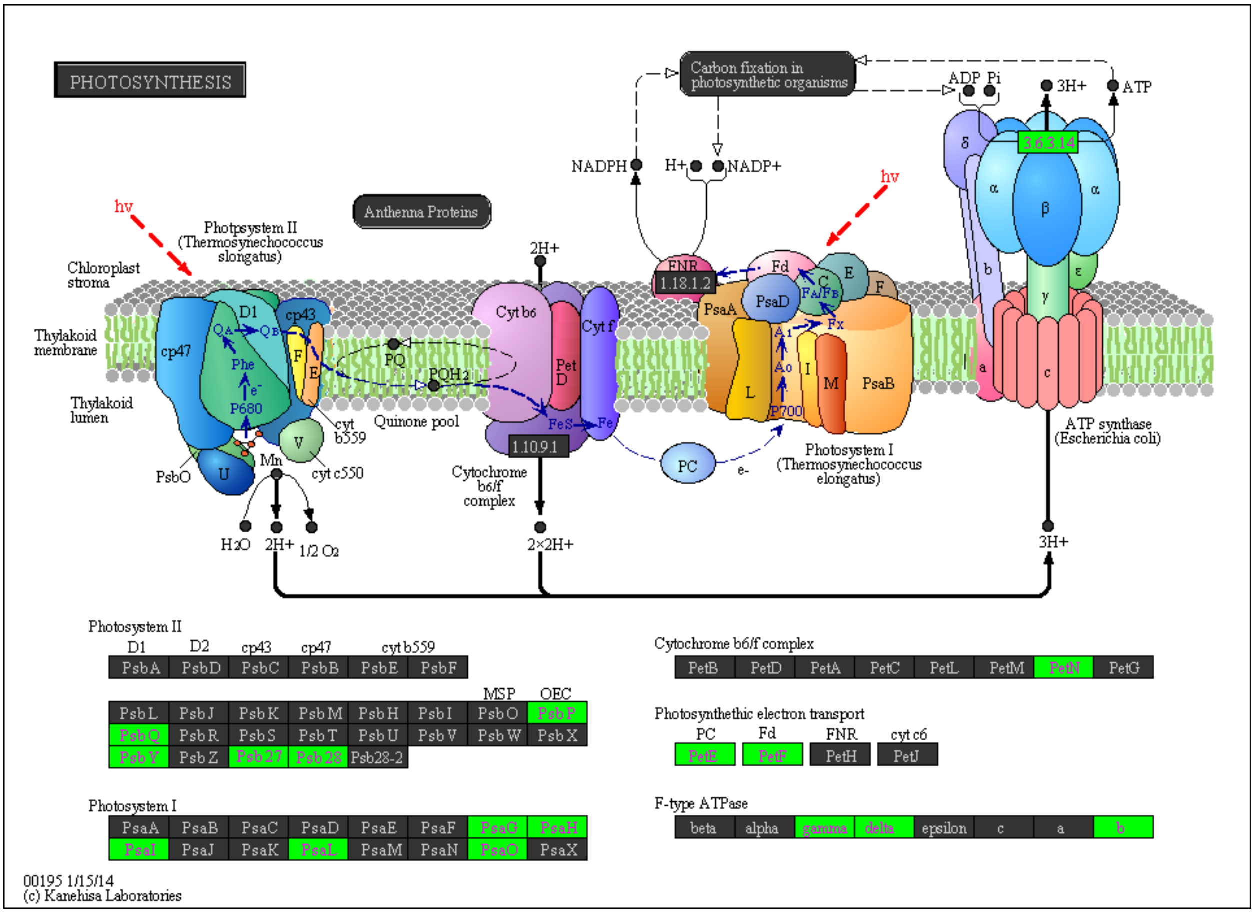

Figure S8. **KEGG pathway analysis of Photosynthesis (Day 5 stationary phase).** Red and green highlighted bars indicate up- (≥ 2) and down- (≤ -2) regulation respectively. PsbP: Photosystem II (PSII) reaction center PsbP family protein (Oxygen-evolving enhancer protein 2), PsbQ: PSII subunit Q-2 (Oxygen-evolving enhancer protein 3), PsbY: PSII Ycf32-related subunit, Psb27/28: PSII assembly proteins, PsaG: Photosystem I (PSI) reaction center subunit G, PsaH: PSI subunit H, PsaI: PSI subunit I, PsaL: PSI subunit L, PsaO: PSI subunit O, PetN: Cytochrome b6-f complex subunit VIII, Cytochrome b6-f complex subunit petN, PetE: Plastocyanin, PetF: 2Fe-2S ferredoxin-like superfamily protein. Images are obtained by KEGG (Kyoto Encyclopedia of Genes and Genomes).

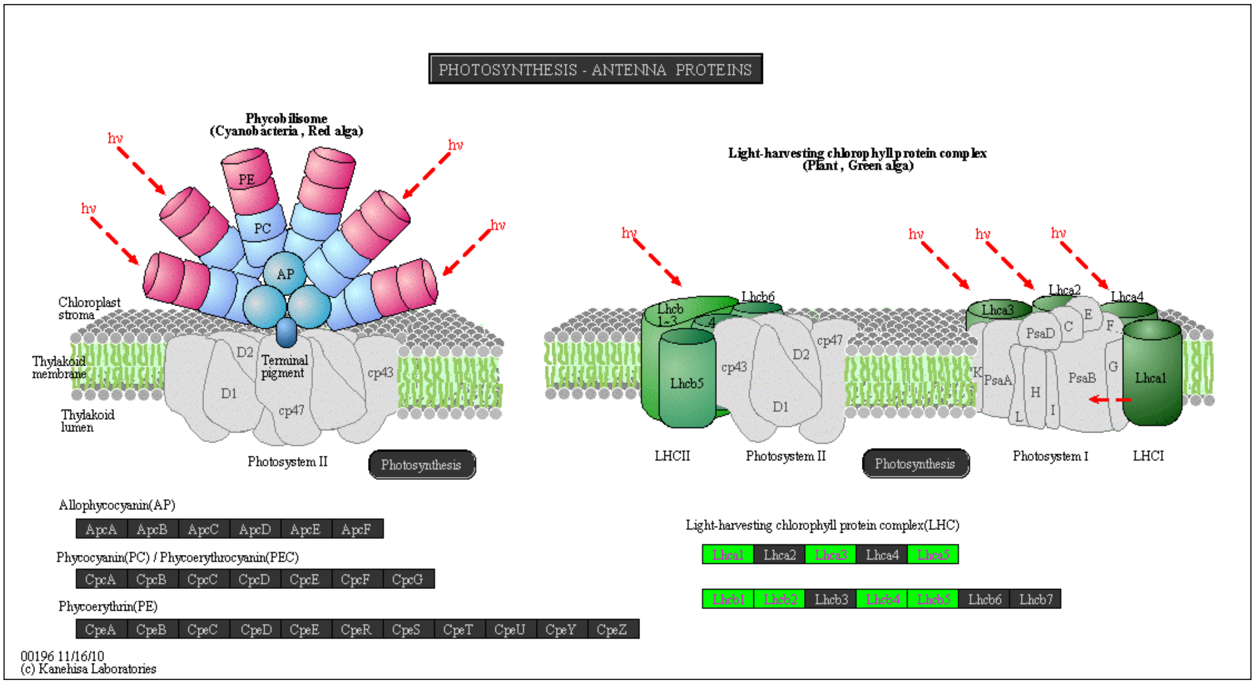

Figure S9. **KEGG pathway analysis of Photosynthesis – Antenna proteins (Day 5 stationary phase).** Red and green highlighted bars indicate up- (≥ 2) and down- (≤ -2) regulation respectively. LHCA1: Photosystem I light harvesting complex (LHC) gene 1, LHCA3: Photosystem I LHC gene 3, LHCA5: Photosystem I LHC gene 5, LHCB1: LHC II chlorophyll a/b binding protein 1, LHCB2: LHC II chlorophyll a/b binding protein 2, LHCB4: LHC II chlorophyll a/b binding protein 4, LHCB5: LHC II chlorophyll a/b binding protein 5. Images are obtained by KEGG (Kyoto Encyclopedia of Genes and Genomes).

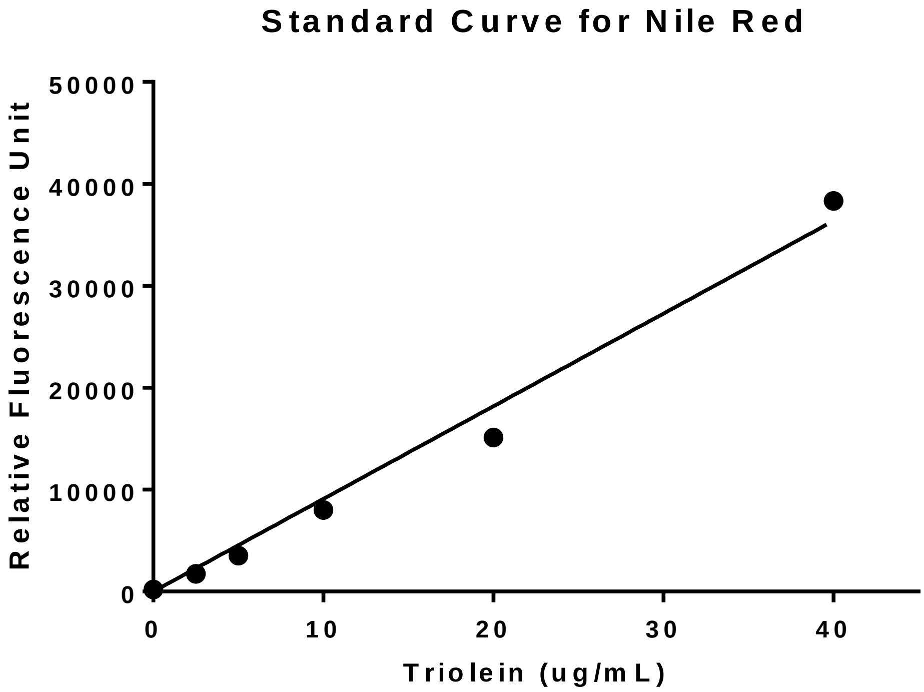

Figure S10. Standard curve for determining neutral lipid concentration. Two hundred microliters of triolein standards (40, 20, 10, 5, 2.5, 0 µg/mL) were loaded as technical triplicates onto a 96-well black, clear bottom plate. Prior to staining, Nile red stock is diluted in acetone to obtain a working solution (25 µg/mL), and 2 µL of the Nile red working solution is added to each well of standard, followed by a 5 min incubation in the dark. Fluorescence of each sample was detected using a microplate reader at excitation and emission wavelengths of 524 nm and 586 nm.

Table S4. Comparison of TAG and starch accumulation in oleaginous and non-oleaginous microalgae.

Species	Carbon source	% TAGs (per DCW)		% Starch (per DCW)		Type of cell	Reference(s)
		N-replete	N-deplete	N-replete	N-deplete		
Dunaliella tertiolecta	Inorganic (CO_2)	0.2%	1%	12.3%	46.1%	High starch; Low TAG	**This Study**
Nannochloropsis oceanica	Inorganic (CO_2)	0.2%	40%	NIL	NIL	High TAG	[37,53]
Chlamydomonas reinhardtii	Organic (Acetate)	14.2%	41.4%	11%	40%	High starch; High TAG	[39–41]
Chlorella vulgaris	Organic (Glucose/acetate glutamate/ lactate)	3.4%	11.5%	23%	50%	High starch; Moderate TAG	[42]

Table S5. Comparison of FA, TAG and starch synthesis genes in oleaginous and non-oleaginous microalgae.

Pathway	Genes	Microalgae species			
		Dunaliella tertiolecta	***Nannochloropsis oceanica***[36]	***Chlamydomonas reinhardtii***[33,43–45]	***Chlorella vulgaris***[10]
**FA synthesis**	***ACCase****	-3.4	-2.6	1.7	-3.0
	MCAT	-4.7	-1.6	1.3	-3.9
	KAS I	-3.6	-1.6	4.1	-4
**TAG synthesis**	***GPAT***	-2.4	0.9		
	LPAAT	3.4	7 isoforms (2 up-, 3 down-, 2 unchanged)	1	2.1
	DGAT		13 isoforms (6 up-, 3 down-, 4 unchanged)	1.7	2.1
	PDAT		1.5	1.4	
**Starch synthesis**	***STA1***	2.4		0.5	2.0
	UGP1	2.6	-3.0		
	SSS1	4.6		1.0	1.6
	SBE2	2.3		0.8	

*ACCase denotes the *Acetyl-CoA biotin carboxyl carrier subunit* as it was the most reported gene among the literature.
